# Synthesis of Bis(Carboranyl)amides 1,1′-μ-(CH_2_NH(O)C(CH_2_)_n_-1,2-C_2_B_10_H_11_)_2_ (*n* = 0, 1) and Attempt of Synthesis of Gadolinium Bis(Dicarbollide)

**DOI:** 10.3390/molecules26051321

**Published:** 2021-03-02

**Authors:** Yasunobu Asawa, Aleksandra V. Arsent’eva, Sergey A. Anufriev, Alexei A. Anisimov, Kyrill Yu. Suponitsky, Oleg A. Filippov, Hiroyuki Nakamura, Igor B. Sivaev

**Affiliations:** 1 School of Life Science and Technology, Tokyo Institute of Technology, Yokohama 226-8501, Japan; asawa.y.aa@m.titech.ac.jp; 2 A.N. Nesmeyanov Institute of Organoelement Compounds, Russian Academy of Sciences, 28 Vavilov Str., 119991 Moscow, Russia; lapppysya@mail.ru (A.V.A.); trueman476@mail.ru (S.A.A.); anisimov.alex.a@gmail.com (A.A.A.); kirshik@yahoo.com (K.Y.S.); h-bond@ineos.ac.ru (O.A.F.); 3 Faculty of Chemical Pharmaceutical Technologies and Biomedical Products, D.I. Mendeleev Russian Chemical Technological University, 9 Miusskaya Sq., 125047 Moscow, Russia; 4 Higher Chemical College at the Russian Academy of Sciences, D.I. Mendeleev Russian Chemical Technological University, 9 Miusskaya Sq., 125047 Moscow, Russia; 5 N.S. Kurnakov Institute of General and Inorganic Chemistry, Russian Academy of Sciences, 31 Leninsky Prosp., 119991 Moscow, Russia; 6 Shemyakin-Ovchinnikov Institute of Bioorganic Chemistry, Russian Academy of Sciences, 16/10 Miklukho- Maklay Str., 117997 Moscow, Russia; 7 Laboratory for Chemistry and Life Science, Institute of Innovative Research, Tokyo Institute of Technology, Yokohama, Kanagawa 226-8503, Japan; hiro@res.titech.ac.jp; 8 Basic Department of Chemistry of Innovative Materials and Technologies, G.V. Plekhanov Russian University of Economics, 36 Stremyannyi Line, 117997 Moscow, Russia

**Keywords:** carboranes, amide derivatives, gadolinium, synthesis, X-ray structure, quantum chemical calculations

## Abstract

Bis(carboranyl)amides 1,1′-μ-(CH_2_NH(O)C(CH_2_)_n_-1,2-C_2_B_10_H_11_)_2_ (*n* = 0, 1) were prepared by the reactions of the corresponding carboranyl acyl chlorides with ethylenediamine. Crystal molecular structure of 1,1′-μ-(CH_2_NH(O)C-1,2-C_2_B_10_H_11_)_2_ was determined by single crystal X-ray diffraction. Treatment of bis(carboranyl)amides 1,1′-μ-(CH_2_NH(O)C(CH_2_)_n_-1,2-C_2_B_10_H_11_)_2_ with ammonium or cesium fluoride results in partial deboronation of the *ortho*-carborane cages to the *nido*-carborane ones with formation of [7,7′(8′)-μ-(CH_2_NH(O)C(CH_2_)_n_-7,8-C_2_B_9_H_11_)_2_]^2−^. The attempted reaction of [7,7′(8′)-μ-(CH_2_NH(O)CCH_2_-7,8-C_2_B_9_H_11_)_2_]^2−^ with GdCl_3_ in 1,2-dimethoxy- ethane did not give the expected metallacarborane. The stability of different conformations of Gd-containing metallacarboranes has been estimated by quantum-chemical calculations using [3,3-μ-DME-3,3′-Gd(1,2-C_2_B_9_H_11_)_2_]^−^ as a model. It was found that in the most stable conformation the CH groups of the dicarbollide ligands are in *anti,anti*-orientation with respect to the DME ligand, while any rotation of the dicarbollide ligand reduces the stability of the system. This makes it possible to rationalize the design of carborane ligands for the synthesis of gadolinium metallacarboranes on their base.

## 1. Introduction

Boron neutron capture therapy (BNCT) is a promising cancer treatment based on the selective accumulation of compounds containing the non-radioactive ^10^B isotope in tumors and their subsequent irradiation with a flux of thermal neutrons. Irradiation leads to the ^10^B(n,α)^7^Li nuclear reaction directly in the cancer cell, and the resulting fission products have a short range comparable to the cell size, which ideally allows the selective destruction of tumor cells without affecting the surrounding healthy tissue [1]. Since the reaction rate is depend on the product of the concentrations of reactants, and the value of the thermal neutron flux is limited by the capabilities of a nuclear reactor or accelerator, one of the most important requirements for BNCT drugs, in addition to their selectivity of accumulation in a tumor, is to achieve a therapeutic concentration of at least 20–35 μg per gram of tumor. This gives rise to an active interest in the use of polyhedral boron hydrides and their derivatives containing 10 or more boron atoms in one molecule as components of BNCT preparations [2,3,4,5,6]. 

Another important condition for effective planning of BNCT treatment of patients is the control of the distribution of the boron-containing drug in the body and its accumulation in the tumor. Since the ^10^B isotope is non-radioactive, its non-invasive determination in the body is a rather difficult task [7,8] and usually requires either the introduction of a radioactive label for positron emission tomography (PET) [9,10], or the measurement of the secondary radiation resulting from the capture of a neutron by the ^10^B nucleus using prompt gamma activation analysis (PGAA) [11]. However, both of these methods require special equipment that is usually not available at medical centers. Therefore, one of the most promising ways to control the distribution of boron-containing drugs in the body and to plan patient treatment is the use of Gd-based magnetic resonance imaging (MRI)—one of the most widely used methods of medical diagnostics [12,13,14]. In addition, the ^157^Gd isotope has the highest thermal neutron capture cross section of all stable nuclides in the periodic table, which exceeds the thermal neutron capture cross section of the ^10^B isotope by more than 60 times. In contrast to ^10^B, the reaction of neutron capture by ^157^Gd induces complex inner shell transitions that generate prompt γ-emission displacing an inner-core electron, which in turn results in internal-conversion electron emission, and finally in the Auger electron emission, together with soft X-ray and photon emission. The path length of Auger electrons in an aqueous medium is only a few nanometers, therefore they have a very high LET and located adjacent to the DNA strands of malignant cells are able to induce a level of DNA injury which is 5–10 times greater than the X-rays, being the most relevant component of gadolinium neutron capture therapy from a radiological point of view [15]. As a result, the presence of gadolinium can lead to a significant increase in the effect of neutron capture therapy when the boron-gadolinium containing drug is localized near the cell nucleus. Therefore, the synthesis of compounds containing both boron and gadolinium is of particular interest for the further development of neutron capture cancer therapy [16]. This interest led to the synthesis of compounds in which the carborane fragment is linked to a Gd-containing DTPA [17,18] or DOTA [19,20,21,22] chelators. Another direction includes synthesis of compounds with the icosahedral *closo*-dodecaborate core carrying multiple Gd^3+^-DOTA-based chelates for MRI [23,24,25].

However, the problem of many Gd-containing complexes is their kinetic stability in vivo. Although at physiological pH 7.4 it is assumed that total excretion of the complexes occurs before any harmful amount of Gd^3+^ is released [26], it has been found that the Gd leakage and retention in body can lead to nephrogenic systemic fibrosis and other health risks [27,28]. Therefore, a real challenge for a boron chemist is to obtain a stable complex in which gadolinium is a part of a metallacarborane cluster, similar to complexes of many other transition metals [29]. In this contribution we describe an attempt to synthesize such a complex based on two *nido*-carborane ligands linked by a flexible spacer containing amide groups.

## 2. Results and Discussion

To the best of our knowledge, there is only one communication in the literature on the synthesis of the gadolinium dicarbollide complex [3,3-(THF)_2_-3,3′-Gd(1,2-C_2_B_9_H_11_)_2_]^−^ [30], however, its characterization is not sufficient to be reliable. At the same time, a careful analysis of the literature on dicarbollide complexes of other lanthanides showed that complexes of this type are usually unstable due to loss of solvent molecules, however their stability can be increased by using carborane ligands with substituents containing pendant donor groups [31]. In this case, the donor groups take the place of easily removable molecules of the coordinated solvent, which leads to an increase in the complex stability due to the chelate effect. Moreover, from the point of view of obtaining the most stable complexes, the best option would be to include these donor groups in a closed chain.

**Figure 1:**
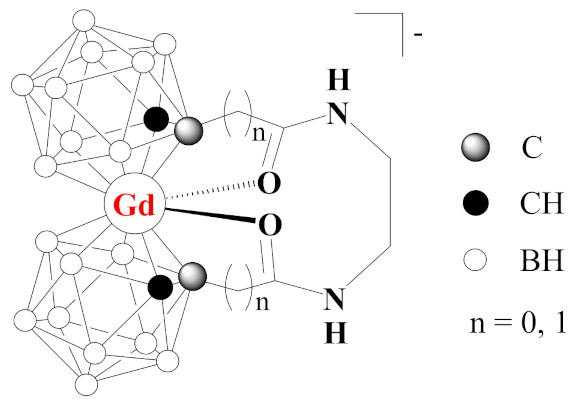
Initial design of Gd-containing metallacarboranes.

Therefore, based on the experience in the synthesis of crowns and thiacrowns with an inserted metallacarborane fragment [32,33], we proposed the design of the ligands in which two *nido*-carborane fragments are linked by a flexible chain containing two amide groups. It was expected that the coordination of gadolinium with such ligands should lead to the metallacarboranes depicted in Figure 1.

To obtain bis(carboranyl)amides, we used well known carboranyl carboxylic acid 1-HOOC-1,2-C_2_B_10_H_11_ (**1**) [34] and carboranyl acetic acid 1-HOOCCH_2_-1,2-C_2_B_10_H_11_ (**2**) [35], which were converted into the corresponding acid chlorides and reacted with ethylenediamine. It should be noted that although the use of carboranyl acid chlorides 1-ClOC(CH_2_)_n_-1,2-C_2_B_10_H_11_ (*n* = 0, 1) for the preparation of amides 1-H_2_N(O)C(CH_2_)_n_-1,2-C_2_B_10_H_11_ was first described more than 50 years ago [36], there are only a few examples of their use in synthesis of the corresponding substituted amides 1-RR’N(O)C(CH_2_)_n_-1,2-C_2_B_10_H_11_ [35,37,38,39], while most of them were obtained either directly by the reaction of 1-lithio-*ortho*-carborane with isocyanates (for amides of carboranyl carboxylic acid) [40,41], or from the corresponding acids using various coupling reagents [42,43,44,45,46,47].

**Scheme 1:**
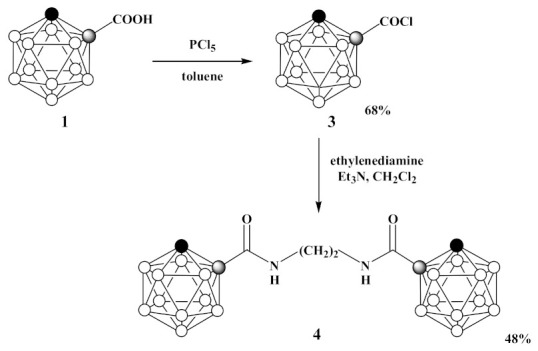
Synthesis of bis(carboranyl)amide 1,1′-μ-(CH_2_NH(O)C-1,2-C_2_B_10_H_11_)_2_ (**4**).

The reaction of *ortho*-carboranyl carboxylic acid chloride 1-Cl(O)C-1,2-C_2_B_10_H_11_ (**3**) with ethylenediamine in dichloromethane in the presence of triethylamine as a base gave the corresponding bis(carboranyl)amide 1,1′-μ-(CH_2_NH(O)C-1,2-C_2_B_10_H_11_)_2_ (**4**) (Scheme 1). The ^1^H-NMR spectrum of bis(carboranyl)amide in acetone-*d*_6_, along with signals of the carborane *CH* and *BH* groups at 4.88 and 3.2–1.4 ppm, respectively, contains signals of the amide NH groups at 7.84 ppm and the ethylene bridge at 3.45 ppm. The ^13^C-NMR spectrum contains signals of the carborane carbon atoms at 59.2 and 73.7 ppm, amide carbonyl groups at 160.3 ppm and the bridging ethylene group at 41.0 ppm. 

**Figure 2:**
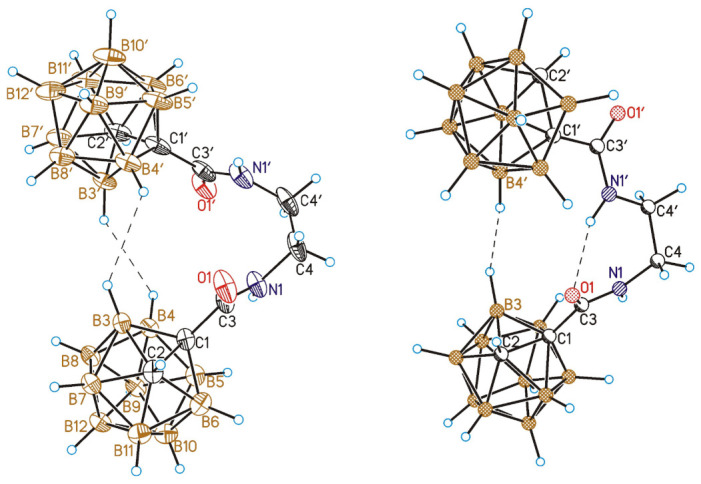
General view of molecular structure of 1,1′-μ-(CH_2_NH(O)C-1,2-C_2_B_9_H_11_)_2_ (**4**) as obtained by X-ray (left) and quantum chemical calculations (right). Experimental view is depicted by thermal ellipsoids at 50% probability. Dashed lines indicate intramolecular noncovalent contacts.

**Figure 3:**
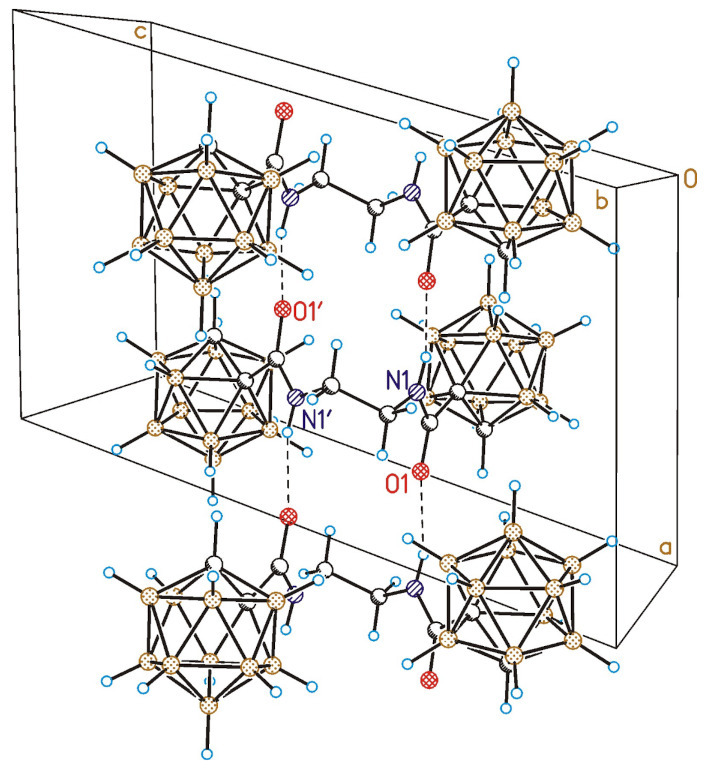
Crystal packing fragment of compound **4** showing H-bonded layers along axis *a*. Solvent molecules are omitted for clarity.

The structure of 1,1′-μ-(CH_2_NH(O)C-1,2-C_2_B_10_H_11_)_2_ (**4**) was determined by single crystal X-ray diffraction (Figure 2). Compound **4** crystallizes in the form of a solvate with molecule of hexane (1:1). The bond lengths in the amide fragments (C_carb_-C(O) 1.527(3) and 1.516(3) Å; C=O 1.231(2) and 1.234(2) Å; C(O)-N 1.332(3) and 1.337(3) Å) are close to those in the known *ortho*-carboranilamides [39,40,48]. The molecular conformation can be described by a set of torsional angles which define geometry of the amide linker and its orientation relative to the cages (Table S1). The observed conformation allows molecules to form H-bonded layers in its crystal structure (Figure 3). It is seen that two intermolecular hydrogen bonds per molecule is formed while no intramolecular hydrogen bonds are observed. To investigate the possibility of the formation of an intramolecular hydrogen bonds in the isolated molecule its structure was optimized using quantum chemical calculations. As expected, N-H…O hydrogen bond (−4.4 kcal/mol) is formed in the isolated molecule (Figure 2) that leads to somewhat different conformation (Table S1). The orientation of the amide groups relative to the adjacent carborane cages changes only slightly while the most significant changes are related to a rotation about N1-C4 (N1′-C4′) bonds. The conformation of the isolated molecule is also stabilized by weak attractive interaction between H3 and H4′ atoms (−0.3 kcal/mol). In somewhat modified way, such interaction is also retained in the crystal (two slightly shorter contacts are observed). 

While for the isolated molecule of **4** the formation of intramolecular hydrogen bond is expected (but only one hydrogen bond can be formed), change of molecular conformation in the crystal structure and, as consequence, absence of this hydrogen bond is explained by formation two intermolecular hydrogen bonds per molecule that would be nearly twice more energetically favorable.

**Scheme 2:**
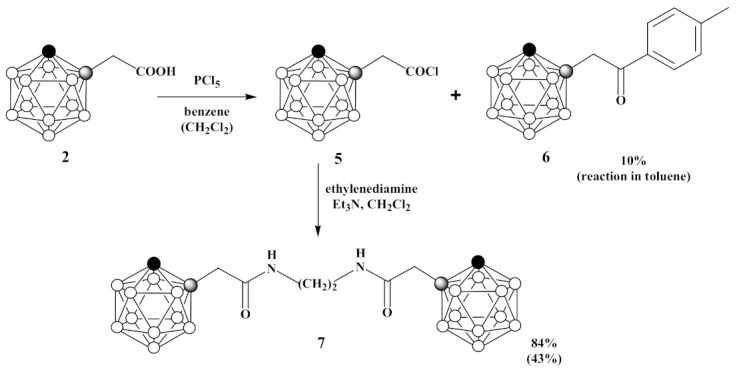
Synthesis of bis(carboranyl)amide 1,1′-μ-(CH_2_NH(O)CCH_2_-1,2-C_2_B_10_H_11_)_2_ (**7**).

Bis(carboranyl)amide 1,1′-μ-(CH_2_NH(O)CCH_2_-1,2-C_2_B_10_H_11_)_2_ (**7**) was prepared by a similar way starting from *ortho*-carboranyl acetic acid. However, in this case, no intermediate purification of the obtained *ortho*-carboranyl acetic acid chloride **5** was carried out, and the crude product was reacted directly with ethylenediamine. When benzene was used in the first stage as a solvent [35], the yield of the final product was 84%, and when trying to replace toxic and environmentally hazardous benzene with dichloromethane, the yield of the bis(carboranyl)amide dropped to 43% (Scheme 2). Attempts to replace benzene with toluene unexpectedly resulted in ketone 1-(*p*-MeC_6_H_4_C(O)CH_2_)- 1,2-C_2_B_10_H_11_ (**6**) as a side-product. Thus, it is obvious that benzene is the best solvent for the preparation of carboranyl carboxylic acid chlorides. The ^1^H-NMR spectrum of 1,1′-(CH_2_NH(O)CCH_2_-1,2-C_2_B_10_H_11_)_2_ (**7**) in acetone-*d*_6_, along with signals of the carborane *CH* and *BH* groups at 4.98 and 3.1–1.3 ppm, contains signals of the amide NH groups at 7.75 ppm, the methylene group of carboranyl acyl fragment at 3.23 ppm and the ethylene bridge at 3.32 ppm. The ^13^C-NMR spectrum contains signals of the carborane carbon atoms at 61.0 and 71.6 ppm, the methylene group of acyl fragment at 39.7 ppm as well as signals of the amide carbonyl groups at 167.4 ppm and the bridging ethylene group at 43.3 ppm.

The ^1^H-NMR spectrum of 1-(*p*-MeC_6_H_4_C(O)CH_2_)-1,2-C_2_B_10_H_11_ (**6**) in acetone-*d*_6_, along with signals of the carborane *CH* group and the methylene group of acyl fragment at 5.08 and 4.12 ppm, respectively, contains characteristic doublets of *para*-substituted benzene at 7.91 and 7.37 ppm as well as the methyl group signal at 2.41 ppm.

**Scheme 3:**
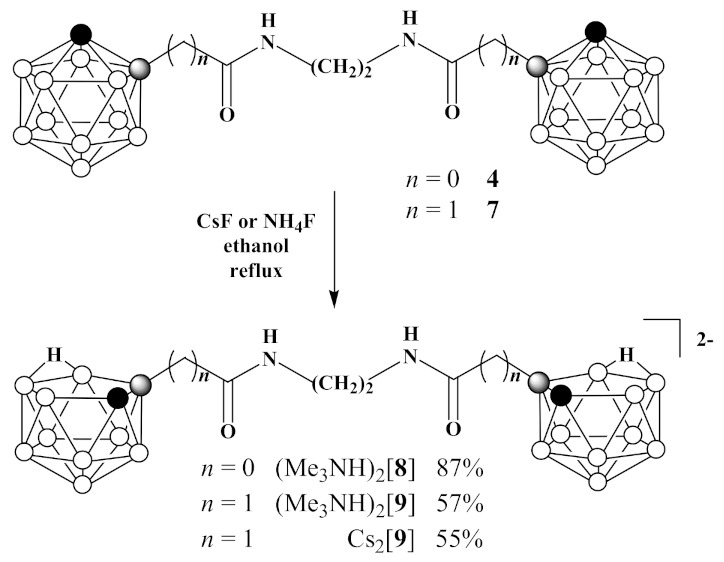
Synthesis of bis(*nido*-carboranyl)amides [7,7′-μ-(CH_2_NH(O)C(CH_2_)_n_-7,8-C_2_B_9_H_11_)_2_]^2−^ (*n* = 0 (**8**), 1 (**9**)).

The treatment of the bis(carboranyl)amides with ammonium or cesium fluoride in refluxing ethanol resulted in partial deboronation of the *closo*-carborane cages with their transformation to the *nido*-carborane ones (Scheme 3). In such way the ligands for synthesis of bis(dicarbollide) complexes were prepared. 

The ^11^B-NMR spectra of the bis(carboranyl) amides [7,7′(8′)-μ-(CH_2_NH(O)C(CH_2_)_n_- 7,8-C_2_B_9_H_11_)_2_]^2−^ (*n* = 0 (L1, **8**), 1 (L2, **9**)) in acetone-*d*_6_ demonstrate the characteristic sets of signals of *C*-substituted *nido*-carborane cage in the range from −9 to −37 ppm. The *closo→nido* transformation of the carborane cage also produces the characteristic high field shifts of the cage carbons and attached to them hydrogen atoms in the ^1^H- and ^13^C-NMR spectra. In addition, in the ^1^H-NMR spectrum of [7,7′(8′)-μ-(CH_2_NH(O)CCH_2_- 7,8-C_2_B_9_H_11_)_2_]^2−^ (**10**) there is the characteristic splitting of the signal of the methylene group, which is directly bonded to the carborane cage, caused by the loss of symmetry of the carborane fragment. 

Unfortunately, our attempts to obtain the expected bis(dicarbollide) gadolinium complex by the reaction of anhydrous gadolinium chloride GdCl_3_ with the bis(carbora- nyl)amide ligand [7,7′(8′)-μ-(CH_2_NH(O)CCH_2_-7,8-C_2_B_9_H_11_)_2_]^2−^ (**9**) in 1,2-dimethoxy- ethane in the presence of *t*-BuOK as a base were unsuccessful. However, we noticed that the mass spectrum of the reaction mixture contains a minor signal at 650.8 corresponding to the [GdL2(DME)]^−^ complex. However, the presence of the DME molecule in the coordination sphere of gadolinium means that it occupies the place that, according to the initial design, should have been occupied by the amide groups of the carborane ligand. In this case, the carborane ligands are most likely rotated relative to each other in another way, and the spacer connecting them, instead of a stabilizing effect on the complexation, on the contrary, can have a destabilizing effect. This prompted us to carry out quantum chemical calculations of the [Gd(C_2_B_9_H_11_)_2_(DME)]^−^ model complex to determine the preferred orientation of the carborane ligands in the case when two sites in the coordination sphere of gadolinium are occupied by a chelate ligand.

It should be noted that, in contrast to the known bis(dicarbollide) complexes of *d*-metals, which in the case of unsubstituted [49] and symmetrically substituted carborane ligands [50,51,52,53] have three nonequivalent stable rotamers, and in the case of asymmetrically substituted ligands [51,52,53,54] have five nonequivalent isomers, the number of possible rotamers in bent bis(dicarbollide) complexes are much higher. This is due to the fact that rotation of the carborane ligands changes not only their relative position relative to each other, but also relative to the chelated DME ligand. 

A large (but probably not exhaustive) set of the [Gd(C_2_B_9_H_11_)_2_(DME)]^−^ rotamers (See Table S2) was obtained by rotating two carborane ligands relative to the DME ligand. For analysis of rotamers two parameters were used—for the carborane ligand rotation respective to DME the dihedral cent(*OO*)-Gd-B_10_-cent(*CC*) (cent is a center of corresponding atoms pair) D1 and D2 for two cages, while the sum of D1 and D2 gives the relative cage rotations D = D1 + D2.

**Figure 4:**
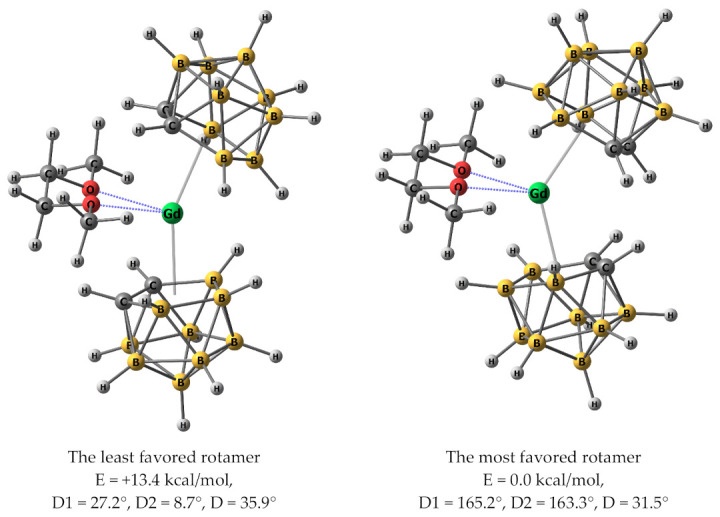
Optimized geometry of the most and the least favored rotamers of gadolinium bis(dicarbollide) [Gd(C_2_B_9_H_11_)_2_(DME)]^−^.

For the four most preferred calculated rotamers, the CH groups of the at least one carborane ligand are in the *anti*-position to the DME ligand (dihedral angle |Di| ≥ 165°), and the most favored rotamer has the CH groups of both ligands in the *anti*-positions to the DME ligand (Figure 4). On the other hand, in the least preferred rotamer the CH groups of both carborane ligands are in the *syn*-positions with respect to the DME ligand (D1 = 27°, D2 = 9°). All the other seven rotamers found have intermediate ligand positions (see Supplementary Materials) typically having one of D1/D2 to be close to 125°. Despite possible errors in the energies which could be up to few kcal/mol, the rather large energy difference between the most favored and second favored rotamers (3.0 kcal/mol), along with large gap between bis-anti and bis-syn rotamers (13.4 kcal/mol) accompanied by consistent dependence of rotamer energy from the D1 and D2 values (increasing of Di favors stabilization) we can state that the carborane ligands undoubtedly prefers *anti* orientation respective to the DME ligand in these complexes.

Thus, the design of the carborane ligands, initially proposed on the basis of purely chemical intuition, was found to be very far from optimal due to the neglect of the optimal rotation of the carborane cages. This leads us to the need to develop a new approach to design of carborane ligands for the synthesis of Gd-containing metallacarboranes, taking into account the optimal rotation of the carborane cages. We hope that this task will be solved in the near future.

## 3. Materials and Methods

### 3.1. General Methods

1-HOOC-1,2-C_2_B_10_H_11_ (**1**) and 1-HOOCCH_2_-1,2-C_2_B_10_H_11_ (**2**) were prepared according to the literature procedures [34,35]. Diethyl ether, tetrahydrofuran, 1,2-dimethoxyethane, dichloromethane, benzene and toluene were dried using standard procedures [55]. All other chemical reagents were purchased from Sigma Aldrich, Acros Organics and ABCR and used without purification. The reaction progress was monitored by thin layer chromatography (Merck F254 silica gel on aluminum plates) and visualized using 0.5% PdCl_2_ in 1% HCl in aq. MeOH (1:10). Acros Organics silica gel (0.060–0.200 mm) was used for column chromatography. The NMR spectra at 400 MHz (^1^H), 128 MHz (^11^B) and 100 MHz (^13^C) were recorded with an Inova 400 spectrometer (Varian). The residual signal of the NMR solvent relative to Me_4_Si was taken as the internal reference for ^1^H- and ^13^C-NMR spectra. ^11^B-NMR spectra were referenced using BF_3_·Et_2_O as external standard. High resolution mass spectra (HRMS) were measured on a micrOTOF II instrument (Bruker) using electrospray ionization (ESI). The measurements were done in a negative ion mode, mass range from *m*/*z* 50 to *m*/*z* 3000. Low resolution mass spectra (MS) were measured on a 2010 EV Liquid Chromatograph-Mass Spectrometer (LC-MS) instrument (Shimadzu) using electrospray ionization (ESI). The measurements were done in a negative mode, mass range from *m*/*z* 200 to *m*/*z* 1000.

### 3.2. Synthesis of 1-Cl(O)C-1,2-C_2_B_10_H_11_ (**3**)

Phosphorus pentachloride (13.50 g, 64.9 mmol) was slowly added to 1-HOOC-1,2-C_2_B_10_H_11_ (**1**) (11.70 g, 62.2 mmol) in 50 mL of toluene and the reaction mixture was stirred for 30 min. Then, the toluene and phosphoryl chloride were removed by distillation at 110 °C. The crude product was purified by vacuum distillation (5 mbar) to collect 1-Cl(O)C-1,2-C_2_B_10_H_11_ (**3**) (8.70 g, yield 68%, bp = 40 °C). 1-Cl(O)C-1,2-C_2_B_10_H_11_ (**3**): ^1^H-NMR (400 MHz, CDCl_3_): δ 4.13 (1H, br s, C*H_Carb_*), 3.4–1.4 (10H, br m, B*H_Carb_*) ppm. ^11^B-NMR (128 MHz, CDCl_3_): δ −1.4 (1B, d, *J* = 108 Hz), −2.1 (1B, d, *J* = 133 Hz), −8.5 (2B, d, *J* = 153 Hz), −11.5 (4B, d, *J* = 187 Hz), −13.2 (2B, d, *J* = 191 Hz) ppm. IR (film): υ_max_ 3074 (CH_Carb_), 2588 (BH_Carb_), 1763 (CO) cm^−1^. The spectral data match those described in the literature [34].

### 3.3. Synthesis of 1,1′-(CH_2_NH(O)C-1,2-C_2_B_10_H_11_)_2_ (**4**)

Under an argon atmosphere a mixture of ethylenediamine (0.10 mL, 1.5 mmol) and triethylamine (0.42 mL, 3.0 mmol) in 10 mL of dichloromethane was added dropwise to 1-Cl(O)C-1,2-C_2_B_10_H_11_ (**3**) (0.62 g, 3.0 mmol) in 10 mL of dichloromethane and reaction mixture was stirred for 60 min. Thereafter, volatiles were removed under reduced pressure. The column chromatography on silica using ethyl acetate as eluent gave pure pale-yellow solid of 1,1′-(CH_2_NH(O)C-1,2-C_2_B_10_H_11_)_2_ (**5**) (0.29 g, yield 48%). ^1^H-NMR (400 MHz, acetone-*d*_6_): δ 7.84 (2H, br s, N*H*), 4.88 (2H, br s, C*H_Carb_*), 3.45 (4H, t (1:1:1), *J*^2^_N,H_ = 2.7 Hz, C*H_2_*), 3.2–1.4 (br m, B*H_Carb_*) ppm. ^11^B-NMR (128 MHz, acetone-*d*_6_): δ −3.9 (4B, d, *J* = 146 Hz), −9.2 (4B, d, *J* = 150 Hz), −12.7 (8B, m), −13.6 (4B, d, *J* = 154 Hz) ppm. ^13^C-NMR (100 MHz, acetone-*d*_6_): δ 160.3 (*C*O), 73.7 (*C_Carb_*CO), 59.2 (*C_Carb_*H), 41.0 (*C*H_2_) ppm. IR (film): υ_max_ 3360 (NH), 3074 (CH_Carb_), 2597 (BH_Carb_), 1684 (CO) cm^−1^. HRMS (ESI): found *m*/*z* 423.4043 [М + Na]**^+^**, C_8_H_28_B_18_N_2_NaO_2_, calculated for C_8_H_28_B_18_N_2_NaO_2_ [М + Na]**^+^** = 423.4054. 

### 3.4. Synthesis of 1-Cl(O)CCH_2_-1,2-C_2_B_10_H_11_ (5), 1,1′-(CH_2_NH(O)CCH_2_-1,2-C_2_B_10_H_11_)_2_ (**6**) and 1-(p-CH_3_C_6_H_4_(O)CCH_2_)-1,2-C_2_B_10_H_11_ (**7**)

(a) Under an argon atmosphere phosphorus pentachloride (1.69 g, 8.2 mmol) was slowly added to 1-HOOCCH_2_-1,2-C_2_B_10_H_11_ (**2**) (1.65 g, 8.2 mmol) in 30 mL of benzene and reaction mixture was stirred for 30 min. Then, the benzene and phosphoryl chloride were removed by distillation at 110 °C. The obtained 1-Cl(O)CCH_2_-1,2-C_2_B_10_H_11_ (**5**) was used in the next step without further purification. Under argon atmosphere mixture of ethylenediamine (0.27 mL, 4.1 mmol) and triethylamine (1.13 mL, 8.2 mmol) in 10 mL of dichloromethane was added dropwise to 1-Cl(O)CCH_2_-1,2-C_2_B_10_H_11_ (**5**) (1.80 g, 8.2 mmol) in 10 mL of dichloromethane and reaction mixture was stirred for 60 min. Thereafter, volatiles were removed under reduced pressure. The column chromatography on silica using ethyl acetate as eluent gave white solid of 1,1′-(CH_2_NH(O)CCH_2_-1,2-C_2_B_10_H_11_)_2_ (**7**) (1.47 g, yield 84%).
(b) Under an argon atmosphere phosphorus pentachloride (0.21 g, 1.0 mmol) was slowly added to 1-HOOCCH_2_-1,2-C_2_B_10_H_11_ (**2**) (0.20 g, 1.0 mmol) in 5 mL of dichloromethane and reaction mixture was stirred for 10 min. Then, the reaction mixture was heated under reflux for 1 h. Thereafter, the dichloromethane and phosphoryl chloride were removed by distillation at 110 °C. The obtained 1-Cl(O)CCH_2_-1,2-C_2_B_10_H_11_ (**5**) was used in the next step without further purification. Under argon atmosphere mixture of ethylenediamine (0.03 mL, 0.5 mmol) and triethylamine (0.14 mL, 1.0 mmol) in 10 mL of dichloromethane was added dropwise to 1-Cl(O)CCH_2_-1,2-C_2_B_10_H_11_ (**5**) in 10 mL of dichloromethane and reaction mixture was stirred for 60 min. Thereafter, volatiles were removed under reduced pressure. The column chromatography on silica using a mixture of ethyl acetate and hexane (1:1, *v*/*v*) as eluent gave pure white solid of 1,1′-(CH_2_NH(O)CCH_2_-1,2-C_2_B_10_H_11_)_2_ (**7**) (0.18 g, yield 43%). 1,1′-(CH_2_NH(O)CCH_2_- 1,2-C_2_B_10_H_11_)_2_ (**7**): ^1^H-NMR (400 MHz, acetone-*d*_6_): δ 7.75 (2H, br s, N*H*), 4.98 (2H, br s, C*H_Carb_*), 3.32 (4H, t (1:1:1), *J*^2^_N,H_ = 2.7 Hz, NC*H_2_*), 3.23 (4H, s, C*H_2_*), 3.1–1.3 (br m, B*H_Carb_*) ppm. ^11^B-NMR (128 MHz, acetone-*d*_6_): δ −2.8 (2B, d, *J* = 147 Hz), −5.6 (2B, d, *J* = 145 Hz), −9.7 (4B, d, *J* = 130 Hz), −10.6 (4B, d, *J* = 137 Hz), −11.7 (4B, d, *J* = 152 Hz), −13.0 (4B, d, *J* = 158 Hz) ppm. ^13^C-NMR (100 MHz, acetone-*d*_6_): δ 167.4 (*C*O), 71.6 (*C_Carb_*CH_2_), 61.0 (*C_Carb_*H), 43.3 (N*C*H_2_), 39.7 (*C*H_2_) ppm. HRMS (ESI): found *m*/*z* 429.4545 [М + H]**^+^**, C_10_H_33_B_20_N_2_O_2_, calculated for C_10_H_33_B_20_N_2_O_2_ [М + H]**^+^** = 429.4549.
(c) Under an argon atmosphere phosphorus pentachloride (0.42 g, 2.0 mmol) was slowly added to 1-HOOCCH_2_-1,2-C_2_B_10_H_11_ (**2**) (0.40 g, 2.0 mmol) in 10 mL of toluene and reaction mixture was stirred for 10 min. Then, the reaction mixture was heated under reflux for 30 min. Thereafter, the toluene and phosphoryl chloride were removed by distillation at 110 °C. A mixture of ethylenediamine (0.07 mL, 1.0 mmol) and triethylamine (0.28 mL, 2.0 mmol) in 10 mL of dichloromethane was added dropwise to solution of crude 1-Cl(O)CCH_2_-1,2-C_2_B_10_H_11_ (1.80 g, 8.2 mmol) in 10 mL of dichloromethane and reaction mixture was stirred for 60 min. Thereafter, volatiles were removed under reduced pressure. The crude material was purified by recrystallization from hexane to give pure colorless crystalline solid of 1-*p*-CH_3_C_6_H_4_(O)CCH_2_-1,2-C_2_B_10_H_11_ (**6**) (0.06 g, yield 10%). ^1^H-NMR (400 MHz, acetone-*d*_6_): δ 7.91 (2H, d, *J* = 8.2 Hz, C*H_Ar_*), 7.37 (2H, d, *J* = 8.2 Hz, C*H_Ar_*), 5.08 (1H, br s, C*H_Carb_*), 4.12 (2H, s, C*H_2_*), 2.41 (3H, s, C*H_3_*) ppm. ^11^B-NMR (128 MHz, acetone-*d*_6_): δ −2.9 (1B, d, *J* = 148 Hz), −5.3 (1B, d, *J* = 146 Hz), −9.7 (2B, d, *J* = 135 Hz), −10.6 (2B, d, *J* = 129 Hz), −11.8 (2B, d, *J* = 158 Hz), −12.9 (2B, d, *J* = 156 Hz) ppm. MS (ESI): found *m*/*z* 275.3 [М − H]^−^, C_11_H_20_B_10_O, calculated for C_11_H_20_B_10_O [М − H]^−^ = 275.2.

### 3.5. Synthesis of (Me_3_NH)_2_[7,7′(8′)-(CH_2_NH(O)C)-7,8-C_2_B_9_H_11_)_2_] ((Me_3_NH)_2_ [**8**])

Ammonium fluoride (0.86 g, 23.3 mmol) was added to 1,1′-(CH_2_NH(O)C-1,2- C_2_B_10_H_11_)_2_ (**4**) (0.47 g, 1.2 mmol) in 30 mL of ethanol and the reaction mixture was heated under reflux until the disappearance of starting material on TLC. Thereafter, volatiles were removed under reduced pressure and to the residue water (10 mL) was added. The aqueous solution was filtered and added to solution of (Me_3_NH)Cl (0.33 g, 3.5 mmol) in 5 mL of water to form precipitate. White solid was filtered and dried over P_2_O_5_ to give (Me_3_NH)_2_[7,7′(8′)-(CH_2_NH(O)C-7,8-C_2_B_9_H_11_)_2_] ((Me_3_NH)_2_[9]) (0.51 g, yield 87%). ^1^H-NMR (400 MHz, acetone-*d*_6_): δ 6.66 (2H, br s, N*H*), 3.18 (4H, s, C*H_2_*), 3.07 (18H, s, Me_3_NH^+^), 2.39 (2H, br s, C*H_Carb_*), 2.8–0.5 (br m, B*H_Carb_*), −2.76 (2H, br q (1:1:1:1), *J* = 44 Hz, B*H*B) ppm. ^11^B-NMR (128 MHz, acetone-*d*_6_): δ −9.5 (2B, d, *J* = 102 Hz), −10.2 (2B, d, *J* = 119 Hz), −14.4 (2B, d, *J* = 154 Hz), −17.3 (4B, d, *J* = 125 Hz), −20.9 (2B, d, *J* = 145 Hz), −23.0 (2B, d, *J* = 150 Hz), −32.6 (2B, dd, *J*_1_ = 132 Hz, *J*_2_ = 44 Hz), −35.8 (2B, d, *J* = 139 Hz) ppm. ^13^C-NMR (100 MHz, acetone-*d*_6_): δ 173.6 (*C*O), 59.7 (*C_Carb_*CO), 45.7 (Me_3_NH^+^), 41.5 (*C_Carb_*H), 40.3 (*C*H_2_) ppm. IR (film): υ_max_ 3410 (NH), 3037 (CH_Carb_), 2528 (BH_Carb_), 1617 (CO) cm^−1^. HRMS (ESI): found *m*/*z* 380.4036 [М + H]^−^, C_8_H_29_B_18_N_2_O_2_, calculated for C_8_H_29_B_18_N_2_O_2_ [М + H]^−^ = 380.4024.

### 3.6. Synthesis of Cs_2_[7,7′(8′)-(CH_2_NH(O)CCH_2_-7,8-C_2_B_9_H_11_)_2_] (Cs_2_ [**9**]) and (Me_3_NH)_2_[7,7′(8′)-(CH_2_NH(O)CCH_2_-7,8-C_2_B_9_H_11_)_2_] ((Me_3_NH)_2_[**9**])

(a) Cesium fluoride (3.12 g, 20.5 mmol) was added to 1,1′-(CH_2_NH(O)CCH_2_-1,2-C_2_B_10_H_11_)_2_ (**7**) (1.47 g, 3.4 mmol) in 30 mL of ethanol and reaction mixture was heated under reflux for 48 h. Thereafter, the reaction mixture was filtered, volatiles were removed under reduced pressure. The column chromatography on silica using ethanol as eluent gave pure white solid of Cs_2_[7,7′(8′)-(CH_2_NH(O)CCH_2_-7,8-C_2_B_9_H_11_)_2_] (Cs_2_[9]) (1.24 g, yield 55%). ^1^H-NMR (400 MHz, acetone-*d*_6_): δ 7.40 (2H, br s, N*H*), 3.40 (4H, s, NC*H_2_*), 2.57 (2H, d, *J* = 14.9 Hz, CH*H*), 2.25 (2H, d, *J* = 14.9 Hz, C*H*H), 1.80 (2H, br s, C*H_Carb_*), −2.79 (2H, br q (1:1:1:1), *J* = 54 Hz, B*H*B) ppm. ^11^B-NMR (128 MHz, acetone-*d*_6_): δ −9.9 (2B, d, *J* = 137 Hz), −11.6 (2B, d, *J* = 151 Hz), −13.8 (4B, d, *J* = 133 Hz), −20.4 (6B, d, *J* = 130 Hz), −32.5 (2B, d, *J* = 118 Hz), −36.5 (2B, d, *J* = 138 Hz) ppm. HRMS (ESI): found *m*/*z* 203.7113 [М]^2−^, C_10_H_32_B_18_N_2_O_2_, calculated for C_10_H_32_B_18_N_2_O_2_ [М]^2−^ = 203.7133.
(b) Ammonium fluoride (0.11 g, 3.0 mmol) was added to 1,1′-(CH_2_NH(O)C-1,2-C_2_B_10_H_11_)_2_ (**7**) (0.09 g, 0.2 mmol) in 30 mL of ethanol and reaction mixture was heated under reflux until the disappearance of starting material on TLC. Thereafter, volatiles were removed under reduced pressure and to the residue water (5 mL) was added. The aquatic solution was filtered and added to trimethylamine hydrochloride (0.10 g, 1.0 mmol) in 5 mL of water to form precipitate. White solid was filtered and dried over P_2_O_5_ to give (Me_3_NH)_2_[7,7′(8′)-(CH_2_NH(O)C-7,8-C_2_B_9_H_11_)_2_] ((Me_3_NH)_2_[9]) (0.06 g, yield 57%). ^1^H-NMR (400 MHz, acetone-*d*_6_): δ 7.15 (br s, N*H*), 3.36 (4H, m, NC*H_2_*), 3.20 (18H, s, Me_3_NH^+^), 2.55 (2H, d, *J* = 15.0 Hz, CH*H*), 2.24 (2H, d, *J* = 15.0 Hz, C*H*H), 1.81 (2H, br s, C*H_Carb_*), −2.71 (2H, br q (1:1:1:1), *J* = 42 Hz, B*H*B) ppm. ^11^B-NMR (128 MHz, acetone-*d*_6_): δ −10.1 (2B, d, *J* = 133 Hz), −11.2 (2B, d, *J* = 138 Hz), −13.7 (2B, d, *J* = 170 Hz), −15.0 (2B, d, *J* = 135 Hz), −19.4 (4B, d, *J* = 130 Hz), −21.0 (2B, d, *J* = 156 Hz), −32.7 (2B, dd, *J*_1_ = 128 Hz, *J*_2_ = 42 Hz), −36.6 (2B, d, *J* = 135 Hz) ppm.

### 3.7. Single Crystal X-ray Diffraction Study

X-ray diffraction experiment for compound **4** was carried out using a SMART APEX2 CCD diffractometer (Bruker) (λ(Mo-K_α_) = 0.71073 Å, graphite monochromator, ϖ-scans) at 120 K. Collected data were processed by the SAINT and SADABS programs incorporated into the APEX2 program package [56]. The structure was solved by the direct methods and re-fined by the full-matrix least-squares procedure against *F*^2^ in anisotropic approximation. The refinement was carried out with the SHELXTL program [57]. Both solvate hexane molecules occupy special position and are disordered over two positions. Occupancy ratios are 0.728(7):0.272(7) and 0.869(9):0.131(9). The CCDC number 2061631 contains the supplementary crystallographic data for this paper. These data can be obtained free of charge via www.ccdc.cam.ac.uk/data_request/cif.

Crystallographic data for **4**: C8H28B20N2O2·C6H14 are triclinic, space group P-1: *a* = 9.6759(11) Å, *b* = 10.3862(12) Å, *c* = 16.0979(17) Å, α = 105.859(3)°, α = 104.585(2)°, γ = 90.158(3)°, *V* = 1501.6(3) Å^3^, *Z* = 1, *M* = 486.69, d_cryst_ = 1.076 g·cm^−3^. *w*R2 = 0.2388 calculated on F^2^_hkl_ for all 7214 independent reflections with 2*θ* < 56.0°, (*GOF* = 1.049, *R* = 0.0763 calculated on F_hkl_ for 4938 reflections with *I* > 2σ(*I*)).

### 3.8. Quantum-Chemical Calculations

For geometry optimization of the [Gd(C_2_B_9_H_11_)_2_(DME)]^−^ complex ORCA v4.2.0 was used [58]. ZORA Hamiltonian [59] was used for treating of relativistic effects, PBE0 functional [60] with Grimme’s DFT-D3 correction [61] and Becke–Johnson damping (D3BJ) [62] was applied. Use of PBE0 functional was proved to be reliable for estimation of geometry and energy of different classes of compounds [63,64,65,66]. The def2-TZVP for light (H, B, C, O) atoms, and scalar all-electron relativistic TZVP basis set (SARC-ZORA- TZVP) [67] for Gd atom were used with the general-purpose auxiliary basis set SARC/J. The RIJCOSX was used to improve the calculations efficiency. The calculations were performed by using an unrestricted formalism (S = 7/2). Such computational approach was shown to lead to a free energy difference of ca. 2–5 kcal/mol in comparison with experiments [68] for lanthanide complexes.

Calculated electron density of compound **4** was analyzed with AIM theory [69] using AIMAll program [70]. Energies of intramolecular noncovalent interactions were estimated using correlation between energy of a contact and potential energy density at bond critical point (*E* = 1/2*V*(*r*)) [71,72] that is widely used for energetic analysis of noncovalent interactions [73,74,75,76].

## Data Availability

The data presented in this study are available in Supplementary Material.

## Supplementary Materials

The following are available online, NMR spectra of compounds **3**–**9**, Table S1: Selected geometry characteristics of compound **4** as obtained from X-ray study and quantum chemical calculation, Table S2: Energies (respective to the most favored isomer in kcal/mol) and principal geometry of [Gd(C_2_B_9_H_11_)_2_(DME)]^−^ rotamers, Figure S1: Optimized geometry of the most favored isomer of [Gd(C_2_B_9_H_11_)_2_(DME)]^−^ (three projections).

## Sample Availability

Samples of the compounds are not available from the authors.

## References

[1] Takagaki M., Kazuko U., Hosmane N.S., Hosmane N.S., Eagling R. (2019). An overview of clinical and biological aspects of current boron neutron capture therapy (BNCT) for cancer treatment. Handbook of Boron Science: With Applications in Organometallics, Catalysis, Materials and Medicine. Volume 4: Boron in Medicine.

[2] Bregadze V.I., Sivaev I.B., Hosmane N.S. (2012). Polyhedral boron compounds for BNCT. Boron Science: New Technologies and Applications.

[3] Sibrian-Vazquez M., Vicente M.G.H., Hosmane N.S. (2012). Boron tumor delivery for BNCT: Recent developments and perspectives. Boron Science: New Technologies and Applications.

[4] Xuan S., Vicente M.G.H., Hey-Hawkins E., Viñas C. (2018). Recent advances in boron delivery agents for boron neutron capture therapy (BNCT). Boron-Based Compounds: Potential and Emerging Applications in Biomedicine.

[5] Ali F., Hosmane N.S., Zhu Y. (2020). Boron chemistry for medical applications. Molecules.

[6] Hu K., Yang Z., Zhang L., Xie L., Wang L., Xu H., Josephson L., Liang S.H., Zhang M.-R. (2020). Boron agents for neutron capture therapy. Coord. Chem. Rev..

[7] Wittig A., Michel J., Moss R.L., Stecher-Rasmussen F., Arlinghaus H.F., Bendel P., Mauri P.L., Altieri S., Hilger R., Salvadori P.A. (2008). Boron analysis and boron imaging in biological materials for Boron Neutron Capture Therapy (BNCT). Critical Rev. Oncol./Hematol..

[8] Protti N., Deagostino A., Boggio P., Alberti D., Geninatti Crich S., Hey-Hawkins E., Viñas C. (2018). New boronated compounds for an imaging-guided personalized neutron capture therapy. Boron-Based Compounds: Potential and Emerging Applications in Biomedicine.

[9] Tolmachev V., Koziorowski J., Sivaev I., Lundqvist H., Carlsson J., Orlova A., Gedda L., Olsson P., Sjöberg S., Sundin A. (1999). *closo*-Dodecaborate(2-) as a linker for iodination of macromolecules. Aspects on conjugation chemistry and biodistribution. Bioconjug. Chem..

[10] Tolmachev V., Bruskin A., Sivaev I., Lundqvist H., Sjöberg S. (2002). Radiobromination of *closo*-dodecaborate anion. Aspects of labelling chemistry in aqueous solution using Chloramine-T. Radiochim. Acta.

[11] Munck af Rosenschöld P.M., Verbakel W.F.A.R., Ceberg C.P., Stecher-Rasmussen F., Persson B.R.R. (2001). Toward clinical application of prompt gamma spectroscopy for in vivo monitoring of boron uptake in boron neutron capture therapy. Med. Phys..

[12] Caravan P., Ellison J.J., McMurry T.J., Lauffer R.B. (1999). Gadolinium(III) chelates as MRI contrast agents: Structure, dynamics, and applications. Chem. Rev..

[13] Yang C.-T., Chuang K.-H. (2012). Gd(III) chelates for MRI contrast agents: From high relaxivity to “smart”, from blood pool to blood–brain barrier permeable. Med. Chem. Commun..

[14] Li X., Sun Y., Ma L., Liu G., Wang Z. (2020). The renal clearable magnetic resonance imaging contrast agents: State of the art and recent advances. Molecules.

[15] Hosmane N.S., Maguire J.A., Zhu Y., Takagaki M. (2012). Boron and Gadolinium Neutron Capture Therapy for Cancer Treatment.

[16] Deagostino A., Protti N., Alberti D., Boggio P., Bortolussi S., Altieri S., Geninatti Crich S. (2016). Insights into the use of gadolinium and gadolinium/boron-based agents in imaging-guided neutron capture therapy applications. Future Med. Chem..

[17] Nemoto H., Cai J., Nakamura H., Fujiwara M., Yamamoto Y. (1999). The synthesis of a carborane gadolinium–DTPA complex for boron neutron capture therapy. J. Organomet. Chem..

[18] Nakamura H., Fukuda H., Girald F., Kobayashi T., Hiratsuka J., Akaizawa T., Nemoto H., Cai J., Yoshida K., Yamamoto Y. (2000). In vivo evaluation of carborane gadolinium-DTPA complex as an MR imaging boron carrier. Chem. Pharm. Bull..

[19] Aime S., Barge A., Crivello A., Deagostino A., Gobetto R., Nervi C., Prandi C., Toppino A., Venturello P. (2008). Synthesis of Gd(III)-C-palmitamidomethyl-C’-DOTAMA-C6-*o*-carborane: A new dual agent for innovative MRI/BNCT applications. Org. Biomol. Chem..

[20] Geninatti-Crich S., Alberti D., Szabo I., Deagostino A., Toppino A., Barge A., Ballarini F., Bortolussi S., Bruschi P., Protti N. (2011). MRI-guided neutron capture therapy by use of a dual gadolinium/boron agent targeted at tumour cells through upregulated low-density lipoprotein transporters. Chem. Eur. J..

[21] Alberti D., Toppino A., Geninatti Crich S., Meraldi C., Prandi C., Protti N., Bortolussi S., Altieri S., Aime S., Deagostino A. (2014). Synthesis of a carborane-containing cholesterol derivative and evaluation as a potential dual agent for MRI/BNCT applications. Org. Biomol. Chem..

[22] Goswami L.N., Cai Q., Ma L., Jalisatgi S.S., Hawthorne M.F. (2015). Synthesis, relaxation properties and in vivo assessment of a carborane-GdDOTA-monoamide conjugate as an MRI blood pool contrast agent. Org. Biomol. Chem..

[23] Goswami L.N., Ma L., Kueffer P.J., Jalisatgi S.S., Hawthorne M.F. (2013). Synthesis and relaxivity studies of a DOTA based nanomolecular chelator assembly supported by an icosahedral *closo*-B_12_^2−^ core for MRI: A click chemistry approach. Molecules.

[24] Goswami L.N., Ma L., Chakravarty S., Cai Q., Jalisatgi S.S., Hawthorne M.F. (2013). Discrete nanomolecular polyhedral borane scaffold supporting multiple gadolinium(III) complexes as a high performance MRI contrast agent. Inorg. Chem..

[25] Goswami L.N., Ma L., Cai Q., Sarma S.J., Jalisatgi S.S., Hawthorne M.F. (2013). cRGD peptide-conjugated icosahedral *closo*-B_12_^2−^ core carrying multiple Gd^3+^-DOTA Chelates for α_v_β_3_ integrin-targeted tumor imaging (MRI). Inorg. Chem..

[26] Port M., Idée J.-M., Medina C., Robic C., Sabatou M., Corot C. (2008). Efficiency, thermodynamic and kinetic stability of marketed gadolinium chelates and their possible clinical consequences: A critical review. BioMetals.

[27] McDonald J.S., McDonald R.J. (2020). MR imaging safety considerations of gadolinium-based contrast agents: Gadolinium retention and nephrogenic systemic fibrosis. Magn. Reson. Imaging Clin. N. Am..

[28] Unruh C., Van Bavel N., Anikovskiy M., Prenner E.J. (2020). Benefits and detriments of gadolinium from medical advances to health and ecological risks. Molecules.

[29] Grimes R.N. (2016). Carboranes.

[30] Lebedev V.N., Shemyakin N.F., Solodovnikov S.P., Zakharkin L.I. (1988). The synthesis of the bis(π-7,8-dicarbaboryl)gadolinium (III) anion. Organomet. Chem. USSR.

[31] Anufriev S.A., Sivaev I.B., Nakamura H. (2020). Two possible ways to combine boron and gadolinium for Gd-guided BNCT. A concept. Phosphorus Sulfur Silicon Relat. Elem..

[32] Teixidor F., Pedrajas J., Rojo I., Viñas C., Kivekäs R., Sillanpää R., Sivaev I., Bregadze V., Sjöberg S. (2003). Chameleonic capacity of [3,3′-Co(1,2-C_2_B_9_H_11_)_2_]^-^ in coordination. Generation of the highly uncommon S(thioether)-Na bond. Organometallics.

[33] Kazakov G.S., Stogniy M.Y., Sivaev I.B., Suponitsky K.Y., Godovikov I.A., Kirilin A.D., Bregadze V.I. (2015). Synthesis of crown ethers with the incorporated cobalt bis(dicarbollide) fragment. J. Organomet. Chem..

[34] Kasar R.A., Knudsen G.M., Kahl S.B. (1999). Synthesis of 3-amino-1-carboxy-*o*-carborane and an improved, general method for the synthesis of all three C-amino-C-carboxycarboranes. Inorg. Chem..

[35] Haushalter R.C., Butler W.M., Rudolph R.W. (1981). The preparation and characterization of several *meso-* tetracarboranylporphyrins. J. Am. Chem. Soc..

[36] Stanko V.I., Klimova A.I., Chapovskii Y.A., Klimova T.P. (1966). Reactions of nucleophilic reagents with esters, ketones, and amides of the carborane series. J. Gen. Chem. USSR.

[37] Kahl S.B., Joel D.D., Finkel G.C., Micca P.L., Nawrocky M.M., Coderre J.A., Slatkin D.N., Fairchild R.G., Bond V.P., Woodhead A.D., Vivirito K. (1989). A carboranyl porphyrin for boron neutron capture therapy of brain tumors. Clinical Aspects of Neutron Capture Therapy.

[38] Evstigneeva R.P., Luzgina V.N., Ol’shevskaya V.A., Zakharkin L.I. (1997). Synthesis of *o*- and *m*-carborane-containing derivatives of 5,10,15,20-tetra-(*p*-aminophenyl)porphyrin. Dokl. Akad. Nauk.

[39] Scholz M., Blobaum A.L., Marnett L.J., Hey-Hawkins E. (2012). *ortho*-Carbaborane derivatives of indomethacin as cyclooxygenase (COX)-2 selective inhibitors. Bioorg. Med. Chem..

[40] Nie Y., Wang Y., Miao J., Bian D., Zhang Z., Cui Y., Sun G. (2014). Synthesis and structural characterization of *o*-carboranylamides with direct cage-amide bond. Dalton Trans..

[41] Xu B., Wang Y.-P., Yao Z.-J., Jin G.-X. (2015). Metal-induced B–H bond activation: Reactions between half-sandwich Ir and Rh complexes with carboranylthioamide. Dalton Trans..

[42] Scholz M.S., Wingen L.M. (2017). Synthesis of dicarba-*closo*-dodecaborane-1-carboxamides. Inorg. Chem..

[43] Orlova A.V., Zinin A.I., Malysheva N.N., Kononov L.O., Sivaev I.B., Bregadze V.I. (2003). Conjugates of polyhedral boron compounds with carbohydrates. 1. New approach to the design of selective agents for boron neutron capture therapy of cancer. Russ. Chem. Bull..

[44] Kononov L.O., Orlova A.V., Zinin A.I., Kimel B.G., Sivaev I.B., Bregadze V.I. (2005). Conjugates of polyhedral boron compounds with carbohydrates. 2. Unexpected easy *closo*- to *nido*-transformation of a carborane–carbohydrate conjugate in neutral aqueous solution. J. Organomet. Chem..

[45] Orlova A.M., Kononov L.O., Kimel B.G., Sivaev I.B., Bregadze V.I. (2006). Conjugates of polyhedral boron compounds with carbohydrates. 4. Hydrolytic stability of carborane–lactose conjugates depends on the structure of a spacer between the carborane cage and sugar moiety. Appl. Organomet. Chem..

[46] Likhosherstov L.M., Novikova O.S., Kononov L.O., Orlova A.V., Sivaev I.B., Bregadze V.I. (2007). Conjugates of polyhedral boron compounds with carbohydrates. 5. Synthesis of glycoconjugates of *closo*-*ortho*-carborane and *N*-acyl-β-lactosylamines with various spacers. Russ. Chem. Bull..

[47] Likhosherstov L.M., Novikova O.S., Kononov L.O., Sivaev I.B., Bregadze V.I. (2009). Conjugates of polyhedral boron compounds with carbohydrates. 6. Synthesis of glycoconjugates of *closo*-*ortho*-carborane with β-lactosylamine and β-D-galactopyranosyl- amine derivatives as galectin bi- and trivalent ligands. Russ. Chem. Bull..

[48] Nie Y., Wang Y., Miao J., Hu C., Zhang Z., Cui Y., Li Y. (2017). Facile deboronation of some *o*-carboranylamides. Eur. J. Inorg. Chem..

[49] Bühl M., Hnyk D., Machaček J. (2005). Computational study of structures and properties of metallaboranes: Cobalt bis(dicarbollide). Chem. Eur. J..

[50] Kazheva O.N., Alexandrov G.G., Kravchenko A.V., Kosenko I.D., Lobanova I.A., Sivaev I.B., Filippov O.A., Shubina E.S., Bregadze V.I., Starodub V.A. (2011). Molecular conductors with a 8-hydroxy cobalt bis(dicarbollide) anion. Inorg. Chem..

[51] Anufriev S.A., Erokhina S.A., Suponitsky K.Y., Godovikov I.A., Filippov O.A., Fabrizi de Biani F., Corsini M., Chizhov A.O., Sivaev I.B. (2017). Methylsulfanyl-stabilized rotamers of cobalt bis(dicarbollide). Eur. J. Inorg. Chem..

[52] Anufriev S.A., Suponitsky K.Y., Filippov O.A., Sivaev I.B. (2019). Synthesis and structure of methylsulfanyl derivatives of nickel bis(dicarbollide). Molecules.

[53] Stogniy M.Y., Anufriev S.A., Shmal’ko A.V., Antropov S.M., Anisimov A.A., Suponitsky K.Y., Filippov O.A., Sivaev I.B. (2021). The unexpected reactivity of 9-iodo-*nido*-carborane: From nucleophilic substitution reactions to the synthesis of tricobalt tris(dicarbollide) Na[4,4′,4”-(MeOCH_2_CH_2_O)_3_-3,3′,3”-Co_3_(μ^3^-O)(μ^3^-S)(1,2-C_2_B_9_H_10_)_3_]. Dalton Trans..

[54] Stogniy M.Y., Kazheva O.N., Chudak D.M., Shilov G.V., Filippov O.A., Sivaev I.B., Kravchenko A.V., Starodub V.A., Buravov L.I., Bregadze V.I. (2020). Synthesis and study of *C*-substituted methylthio derivatives of cobalt bis(dicarbollide). RSC Adv..

[55] Armarego W.L.F., Chai C.L.L. (2009). Purification of Laboratory Chemicals.

[56] (2014). APEX2 and SAINT.

[57] Sheldrick G.M. (2015). Crystal structure refinement with SHELXL. Acta Cryst. C.

[58] Neese F. (2017). Software update: The ORCA program system, version 4.0. WIREs Comp. Mol. Sci..

[59] van Wüllen C. (1998). Molecular density functional calculations in the regular relativistic approximation: Method, application to coinage metal diatomics, hydrides, fluorides and chlorides, and comparison with first-order relativistic calculations. J. Chem. Phys..

[60] Adamo C., Barone V. (1999). Toward reliable density functional methods without adjustable parameters: The PBE0 model. J. Chem. Phys..

[61] Grimme S., Antony J., Ehrlich S., Krieg H. (2010). A consistent and accurate ab initio parametrization of density functional dispersion correction (DFT-D) for the 94 elements H-Pu. J. Chem. Phys..

[62] Grimme S., Ehrlich S., Goerigk L. (2011). Effect of the damping function in dispersion corrected density functional theory. J. Comput. Chem..

[63] Medvedev M.G., Bushmarinov I.S., Sun J.W., Perdew J.P., Lyssenko K.A. (2017). Density functional theory is straying from the path toward the exact functional. Science.

[64] Suponitsky K.Y., Burakov N.I., Kanibolotsky A.L., Mikhailov V.A. (2016). Multiple noncovalent bonding in halogen complexes with oxygen organics. I. Tertiary amides. J. Phys. Chem. A.

[65] Suponitsky K.Y., Tafur S., Masunov A.E. (2008). Applicability of hybrid density functional theory methods to calculation of molecular hyperpolarizability. J. Chem. Phys..

[66] Suponitsky K.Y., Masunov A.E., Antipin M.Y. (2008). Conformational dependence of the first molecular hyperpolarizability in the computational design of nonlinear optical materials for optical switching. Mendeleev. Commun..

[67] Pantazis D.A., Neese F. (2009). All-electron scalar relativistic basis sets for the lanthanides. Chem. Theory Comput..

[68] Jaoul A., Nocton G., Clavaguéra C. (2017). Assessment of density functionals for computing thermodynamic properties of lanthanide complexes. Chem. Phys. Chem..

[69] Bader R.F.W. (1990). Atoms in Molecules. A Quantum Theory.

[70] Keith T.A. (2015). AIMAll (Version 15.05.18).

[71] Espinosa E., Molins E., Lecomte C. (1998). Hydrogen bond strengths revealed by topological analyses of experimentally observed electron densities. Chem. Phys. Lett..

[72] Espinosa E., Alkorta I., Rozas I., Elguero J., Molins E. (2001). About the evaluation of the local kinetic, potential and total energy densities in closed-shell interactions. Chem. Phys. Lett..

[73] Dalinger I.L., Serushkina O.V., Muravyev N.V., Meerov D.B., Miroshnichenko E.A., Kon’kova T.S., Suponitsky K.Y., Vener M.V., Sheremetev A.B. (2018). Azasydnone—novel “green” building block for designing high energetic compounds. J. Mater. Chem. A.

[74] Sheremetev A.B., Aleksandrova N.S., Semyakin S.S., Suponitsky K.Y., Lempert D.B. (2019). Synthesis and characterization of 3-(5-(fluorodinitromethyl)-1H-1,2,4-triazol-3-yl)-4-nitrofurazan: A novel promising energetic component of boron-based fuels for rocket ramjet engines. Chem. Asian J..

[75] Dmitrienko A.O., Karnoukhova V.A., Potemkin A.A., Struchkova M.I., Kryazhevskikh I.A., Suponitsky K.Y. (2017). The influence of halogen type on structural features of compounds containing α-halo-α,α-dinitroethyl moieties. Chem. Heterocycl. Comp..

[76] Anufriev S.A., Sivaev I.B., Suponitsky K.Y., Godovikov I.A., Bregadze V.I. (2017). Synthesis of 10-methylsulfide and 10-alkylmethylsulfonium *nido*-carborane derivatives: B-H···π Interactions between the B-H-B hydrogen atom and alkyne group in 10-RC≡CCH_2_S(Me)-7,8-C_2_B_9_H_11_. Eur. J. Inorg. Chem..

