# Supplementary material for: Synthesis of Bis(Carboranyl)amides 1,1′-μ-(CH2NH(O)C(CH2)n-1,2-C2B10H11)2 (n = 0, 1) and Attempt of Synthesis of Gadolinium Bis(Dicarbollide)
Source: Molecules. 2021 Mar 2;26(5):1321. doi: 10.3390/molecules26051321 (PMC7958119; doi:10.3390/molecules26051321)
Supplement: Supplementary file 1 [file molecules-26-01321-s001.zip › Gd+Carborane-SI(Rev).pdf]

SUPPORTING INFORMATION

Synthesis of Bis(Carboranyl)amides 1,1′-μ-($CH_2NH(O)C(CH_2)_n$-1,2-$C_2B_{10}H_{11})_2$ (n = 0, 1) and Attempt of Synthesis of Gadolinium Bis(Dicarbollide)

Yasunobu Asawa [1], Aleksandra V. Arsent'eva [2,3], Sergey A. Anufriev [2], Alexei A. Anisimov [2,4], Kyrill Yu. Suponitsky [2,5], Oleg A. Filippov [2,6], Hiroyuki Nakamura [7] and Igor B. Sivaev [2,8*]

[1] School of Life Science and Technology, Tokyo Institute of Technology, Yokohama, 226-8501, Japan; asawa.y.aa@m.titech.ac.jp (Y.A.)
[2] A.N. Nesmeyanov Institute of Organoelement Compounds, Russian Academy of Sciences, 28 Vavilov Str., 119991 Moscow, Russia; lapppysya@mail.ru (A.V.A.); trueman476@mail.ru (S.A.A.); anisimov.alex.a@gmail.com (A.A.A.); kirshik@yahoo.com (K.Y.S.); h-bond@ineos.ac.ru (O.A.F.); sivaev@ineos.ac.ru (I.B.S.)
[3] Faculty of Chemical Pharmaceutical Technologies and Biomedical Products, D.I. Mendeleev Russian Chemical Technological University, 9 Miusskaya Sq., 125047 Moscow, Russia;
[4] Higher Chemical College at the Russian Academy of Sciences, D.I. Mendeleev Russian Chemical Technological University, 9 Miusskaya Sq., 125047 Moscow, Russia;
[5] N.S. Kurnakov Institute of General and Inorganic Chemistry, Russian Academy of Sciences, 31 Leninsky Prosp., 119991, Moscow, Russia;
[6] Shemyakin-Ovchinnikov Institute of Bioorganic Chemistry, Russian Academy of Sciences, 16/10 Miklukho- Maklay Str., 117997, Moscow, Russia;
[7] Laboratory for Chemistry and Life Science, Institute of Innovative Research, Tokyo Institute of Technology, Yokohama, Kanagawa 226-8503, Japan; hiro@res.titech.ac.jp (H.N.)
[8] Basic Department of Chemistry of Innovative Materials and Technologies, G.V. Plekhanov Russian University of Economics, 36 Stremyannyi Line, 117997 Moscow, Russia
* Correspondence: sivaev@ineos.ac.ru

NMR spectra of compound **3** — **2**

NMR spectra of compound **4** — **5**

NMR spectra of compound **6** — **10**

NMR spectra of compound **7** — **13**

NMR spectra of compound **$(Me_3NH)_2$[8]** — **18**

NMR spectra of compound **$(Me_3NH)_2$[9]** — **23**

NMR spectra of compound **Cs_2[9]** — **26**

Geometry characteristics of compound **4** — **29**

Geometry and energies of $[Gd(C_2B_9H_{11})_2(DME)]^-$ rotamers — **30**

^{1}H NMR, Compound 3

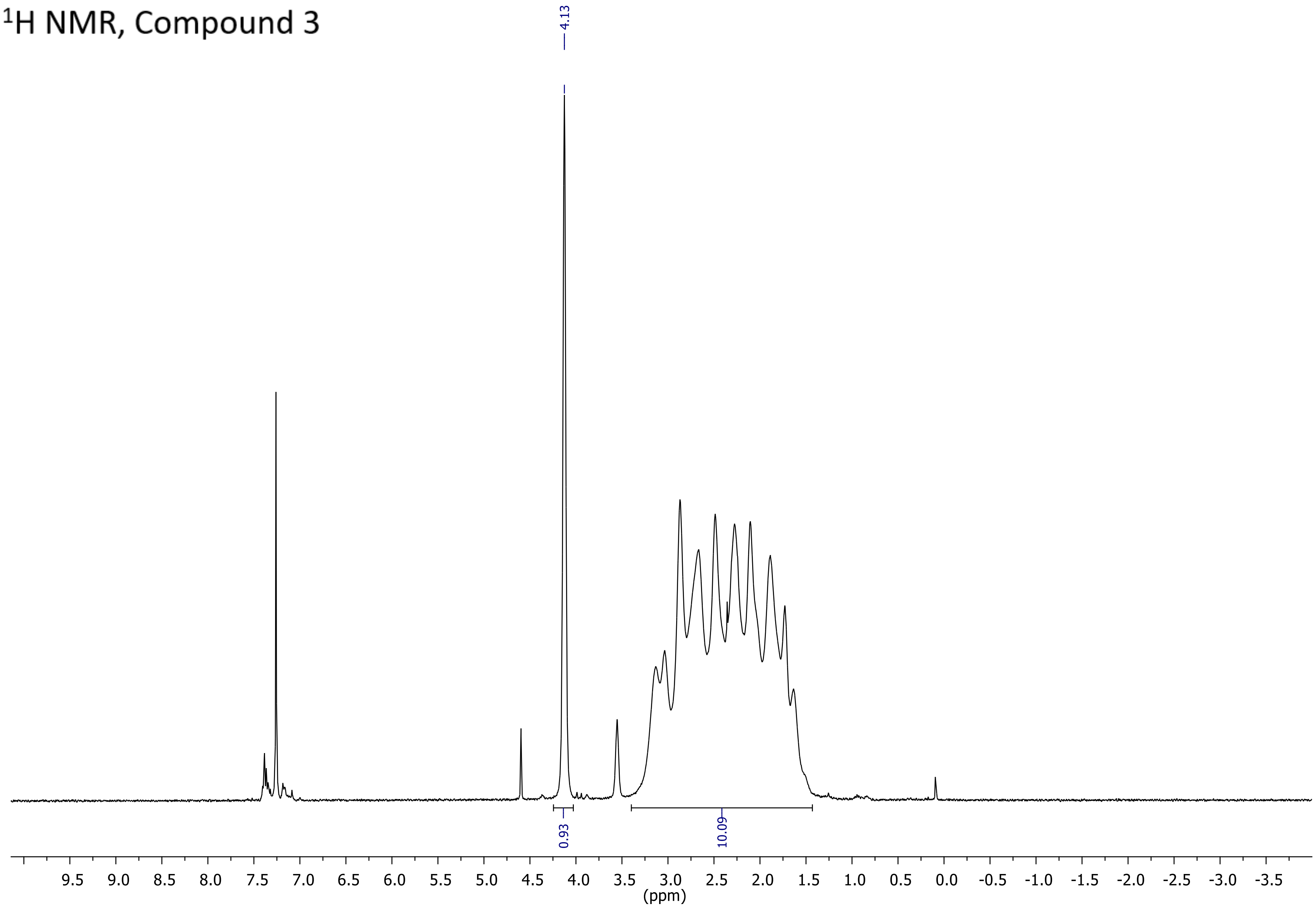

$^{11}B\{^{1}H\}$ NMR, Compound 3

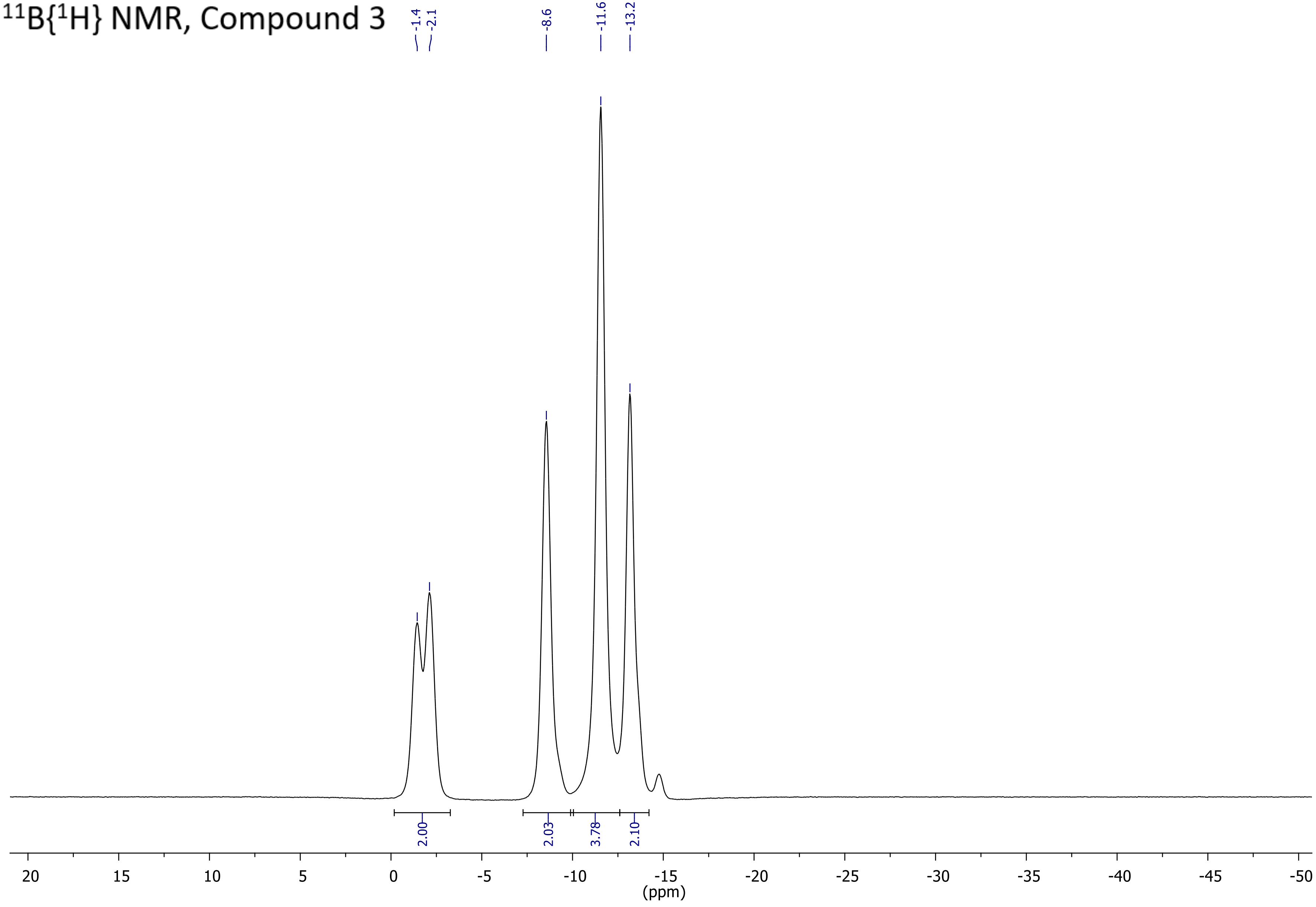

^{11}B NMR, Compound 3

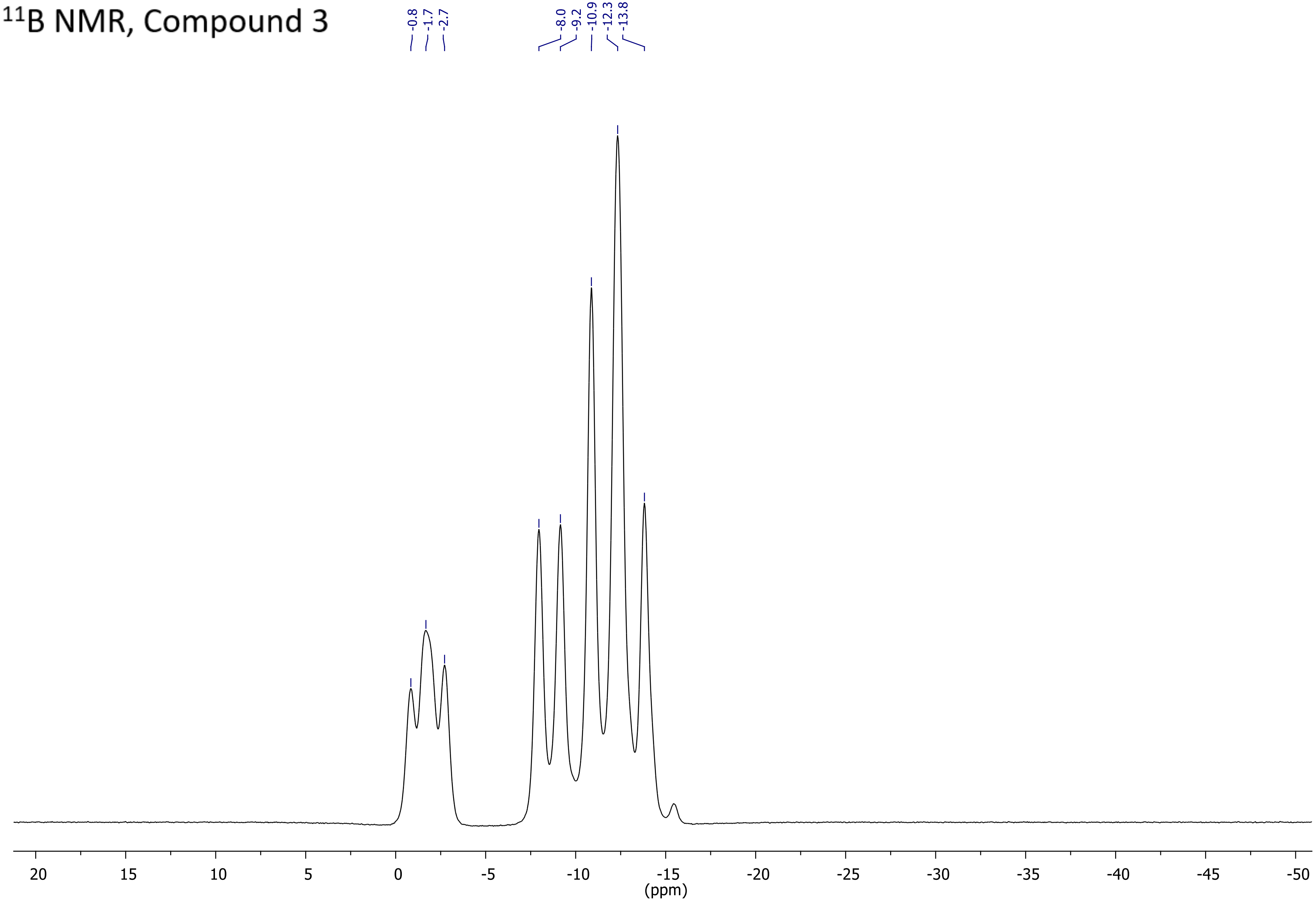

^{1}H NMR, Compound 4

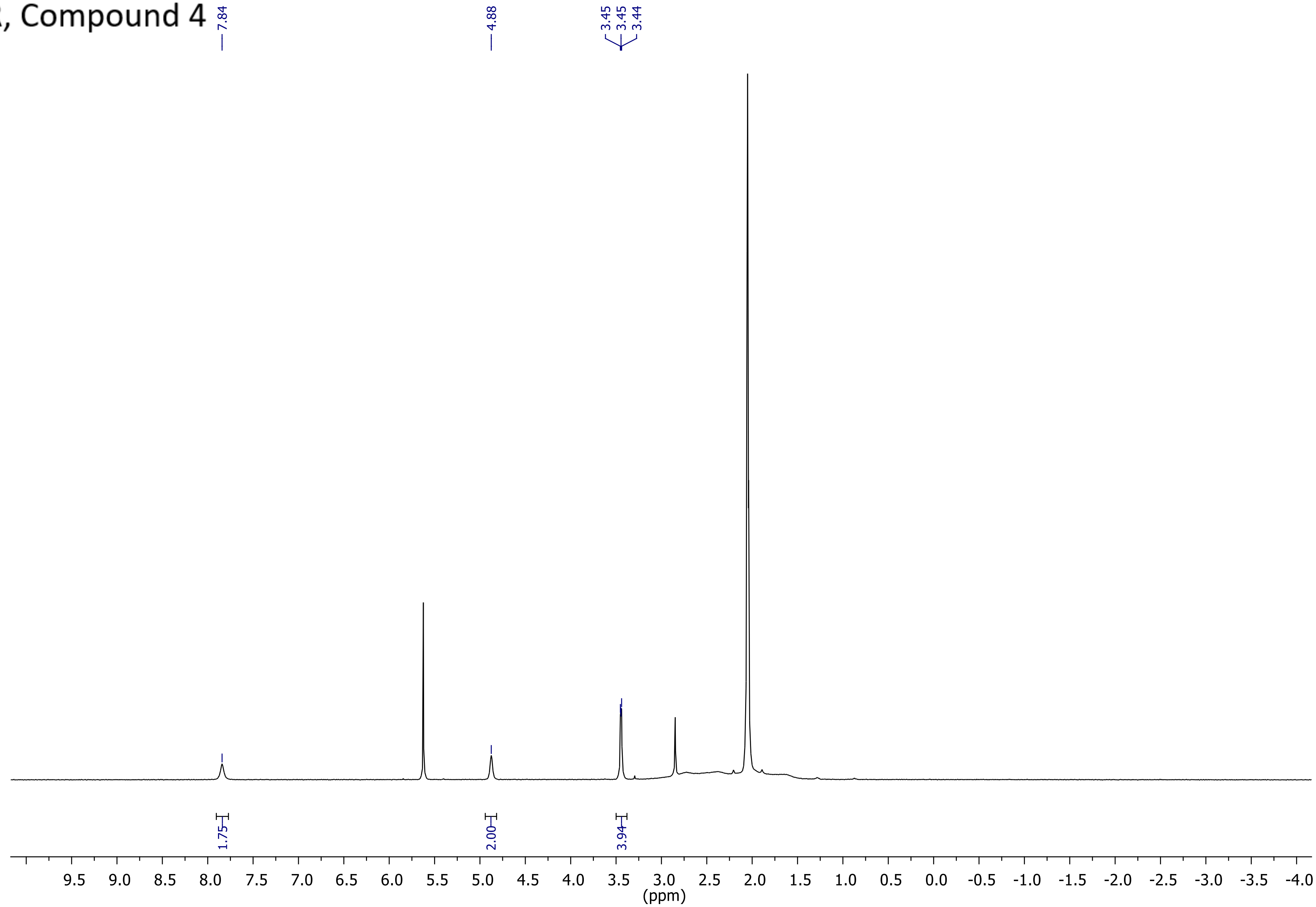

$^{11}B\{^{1}H\}$ NMR, Compound 4

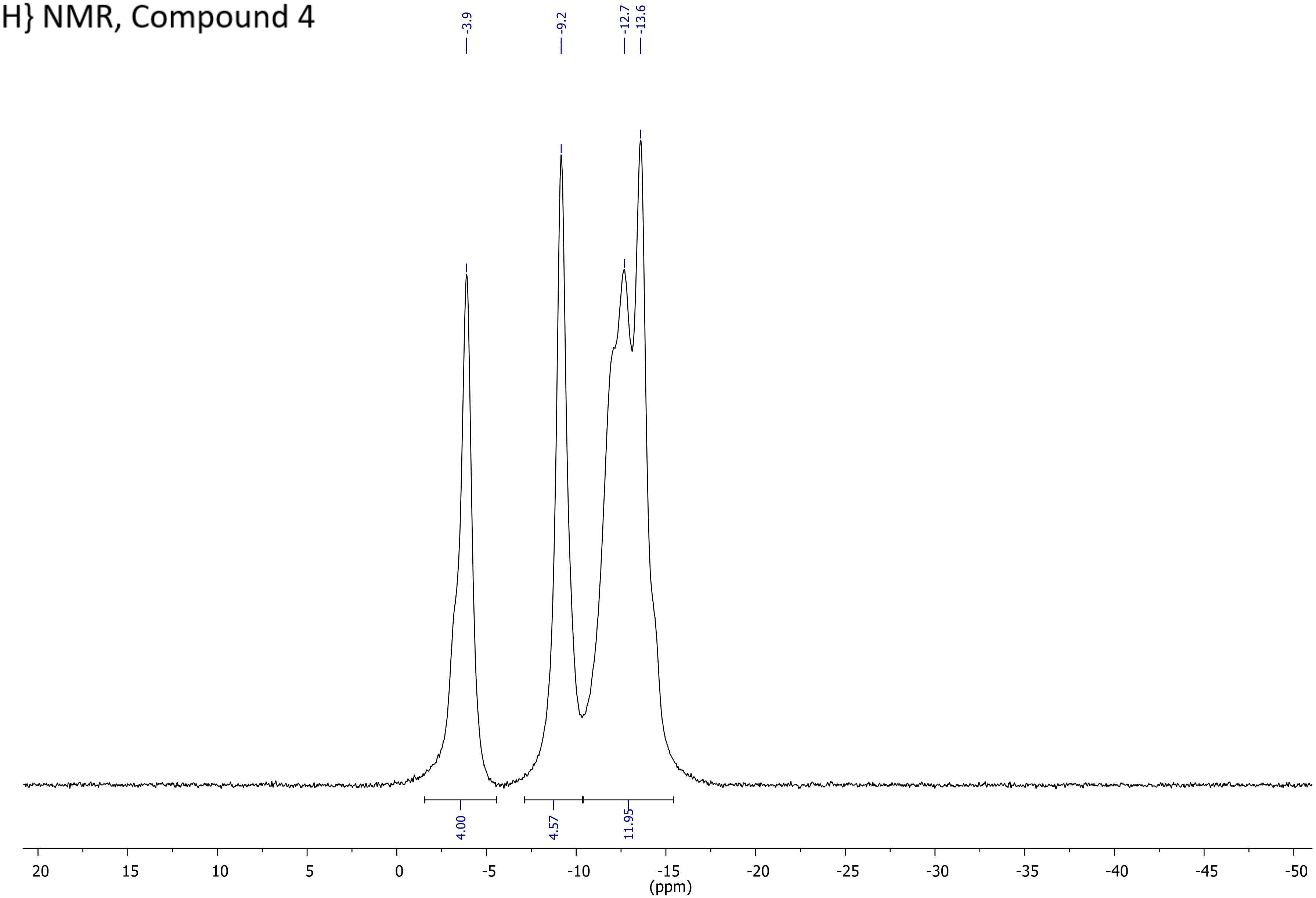

^{11}B NMR, Compound 4

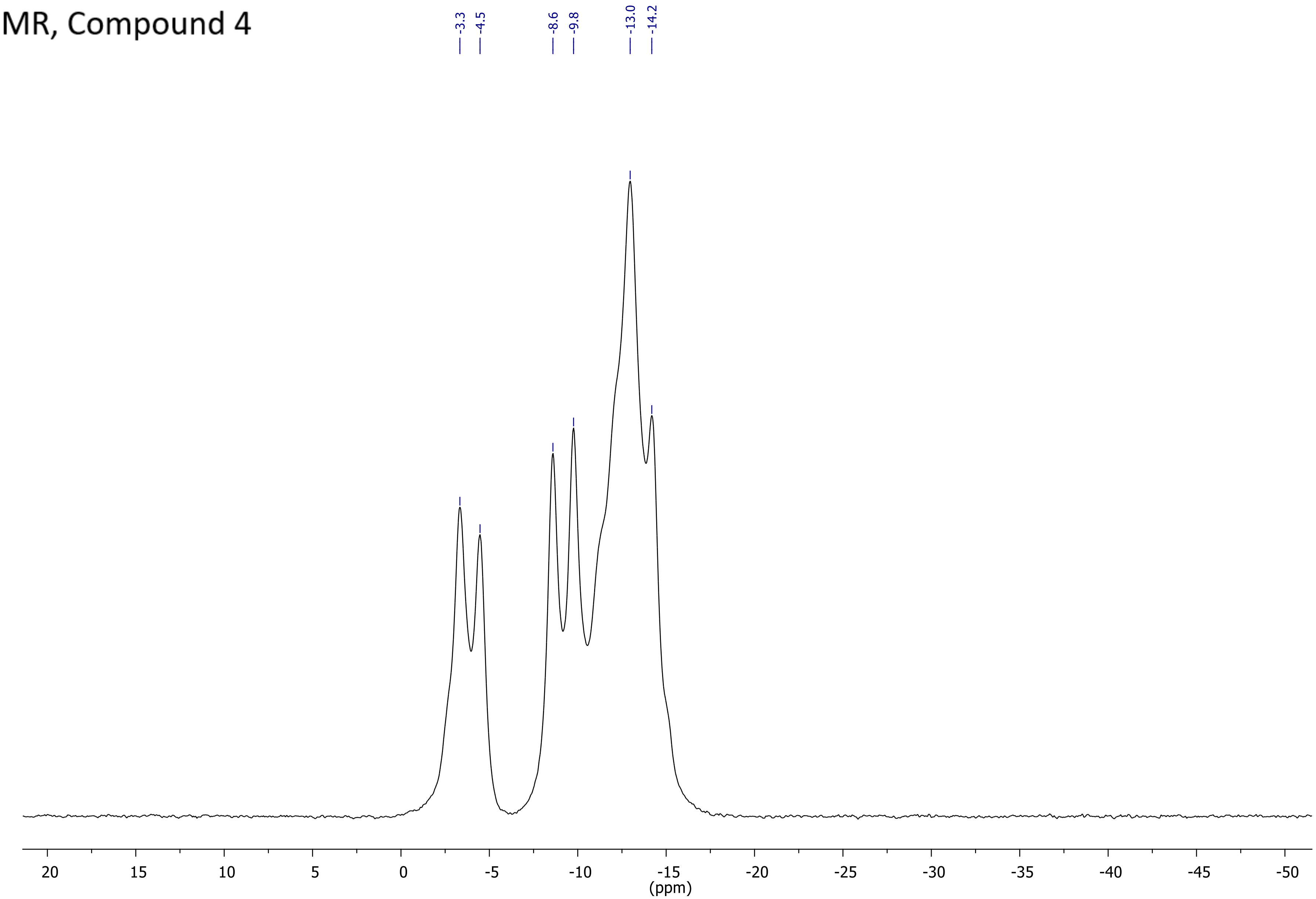

$^{13}C\{^1H\}$ NMR, Compound 4

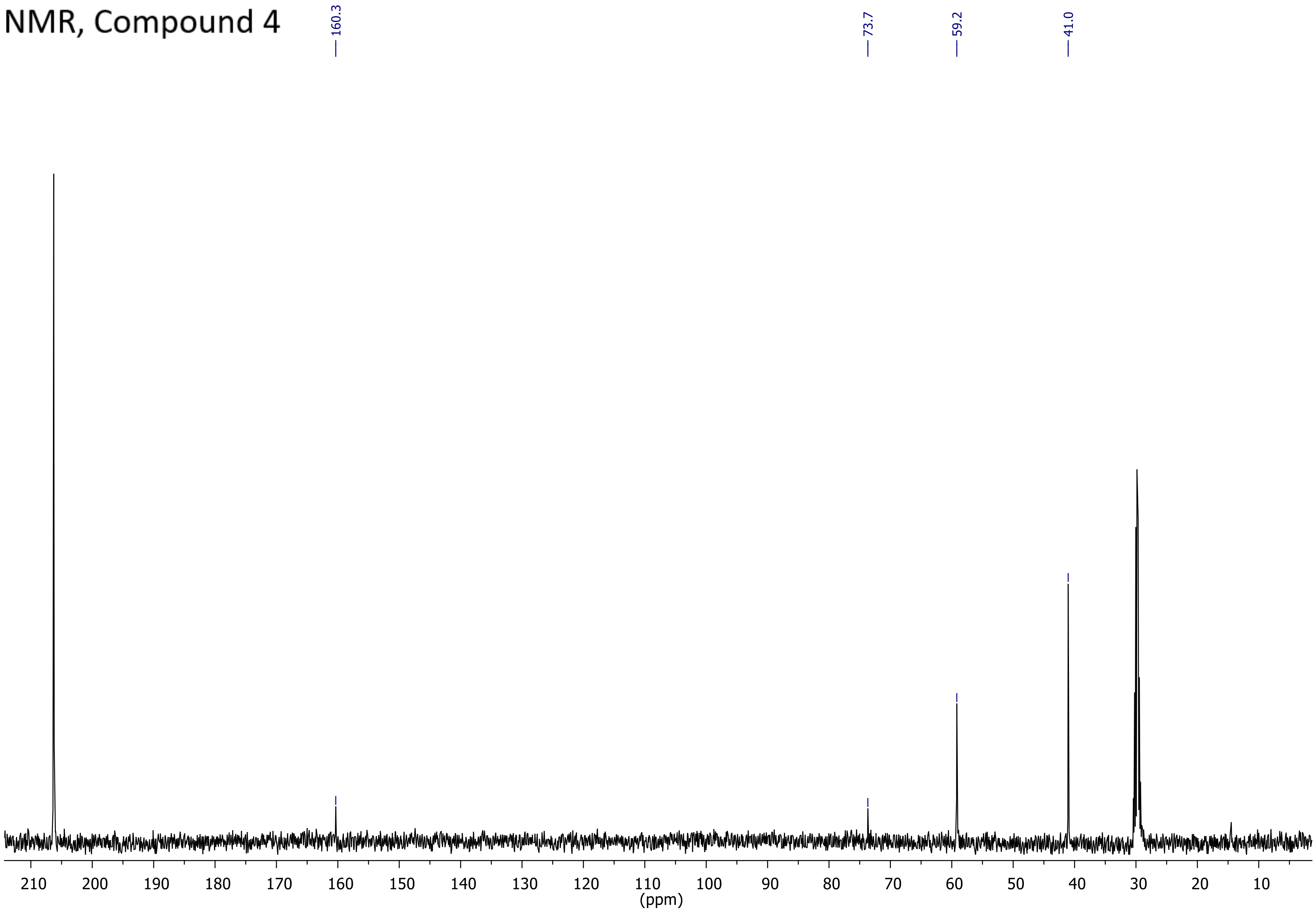

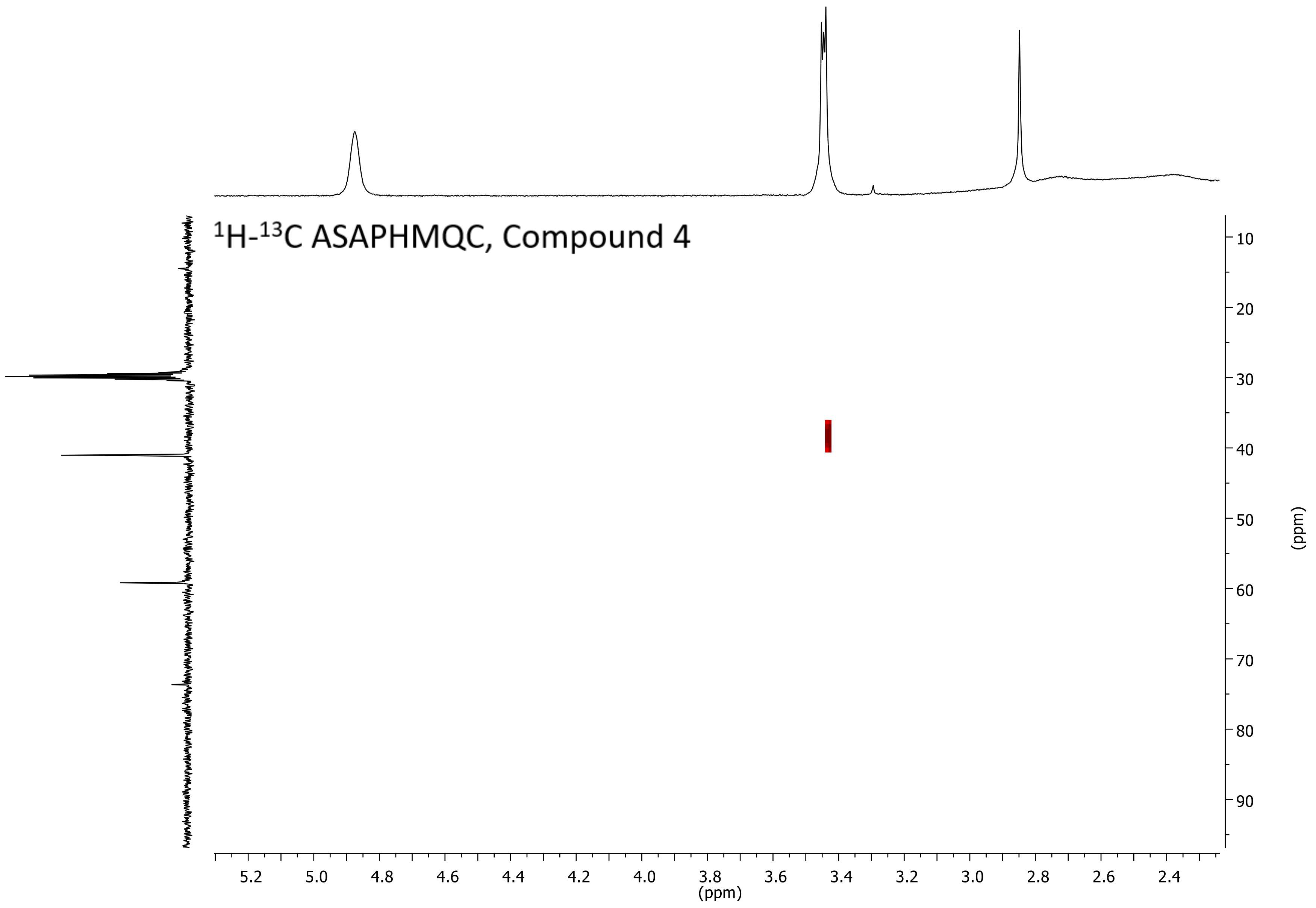

^{1}H-^{13}C ASAPHMQC, Compound 4
5.2
5.0
4.8
4.6
4.4
4.2
4.0
3.8
3.6
3.4
3.2
3.0
2.8
2.6
2.4
(ppm)
10
20
30
40
50
60
70
80
90
(ppm)

^{1}H NMR, Compound 6

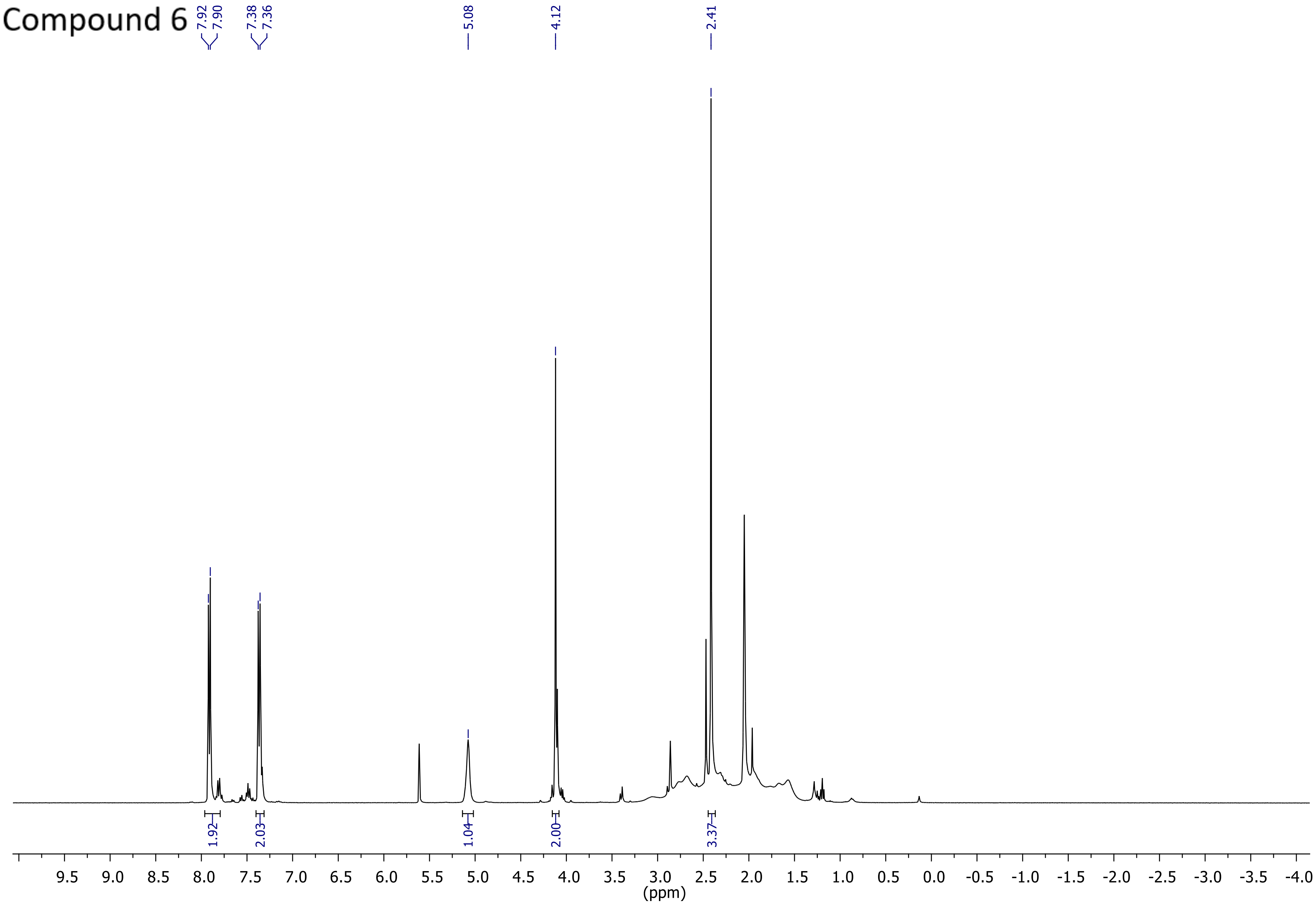

^{11}B NMR, Compound 6

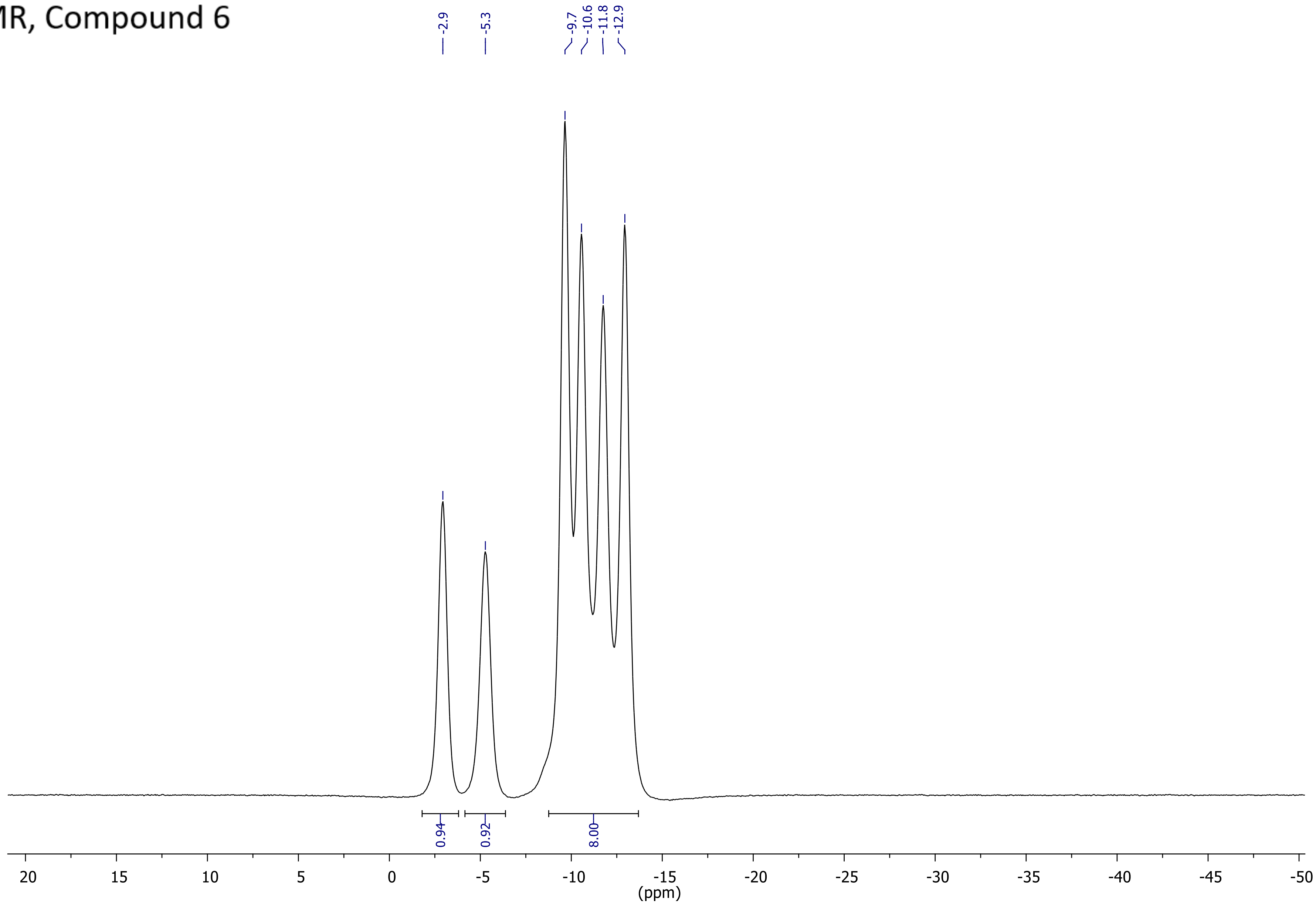

$^{11}B\{^1H\}$ NMR, Compound 6

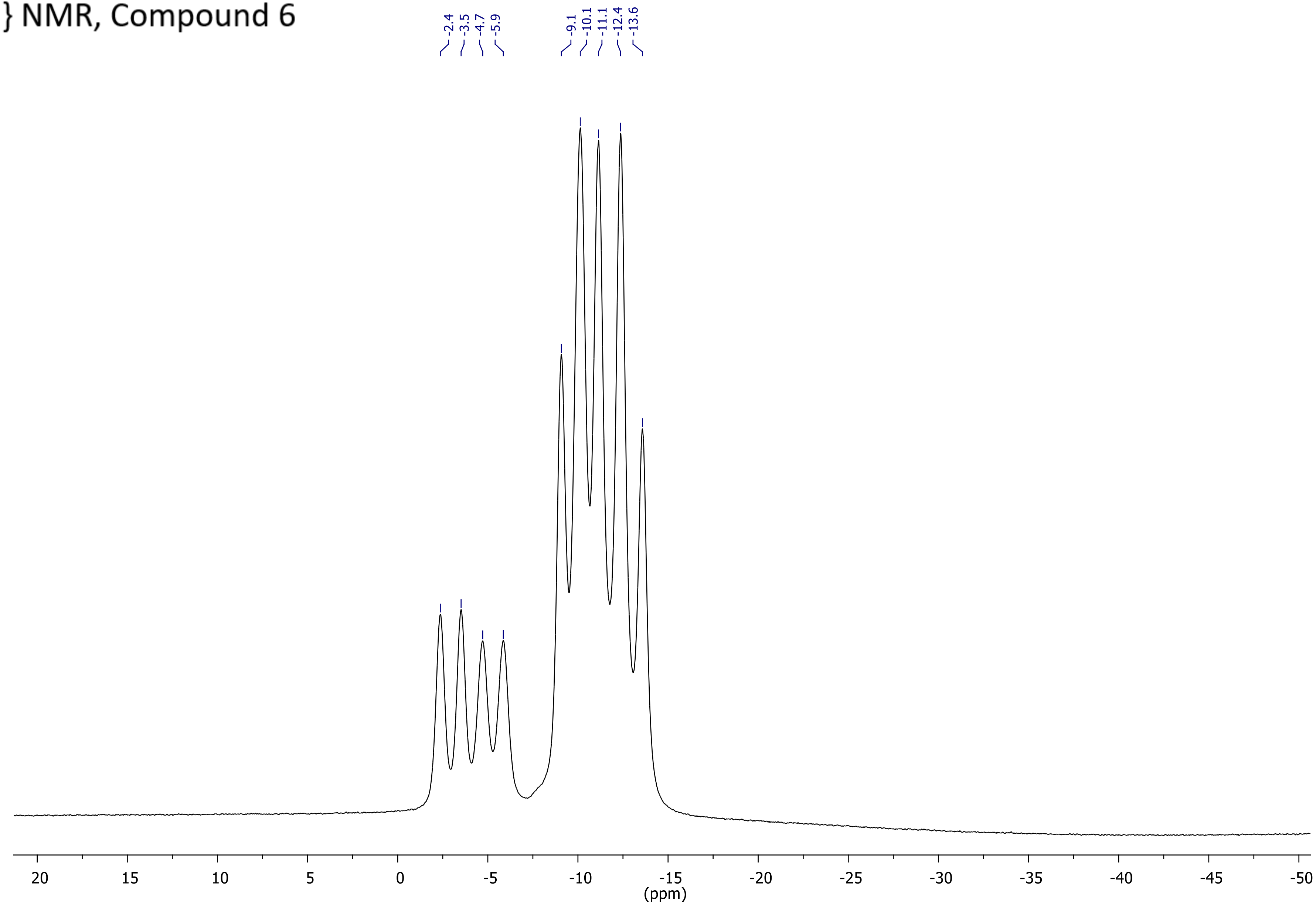

^{1}H NMR, Compound 7

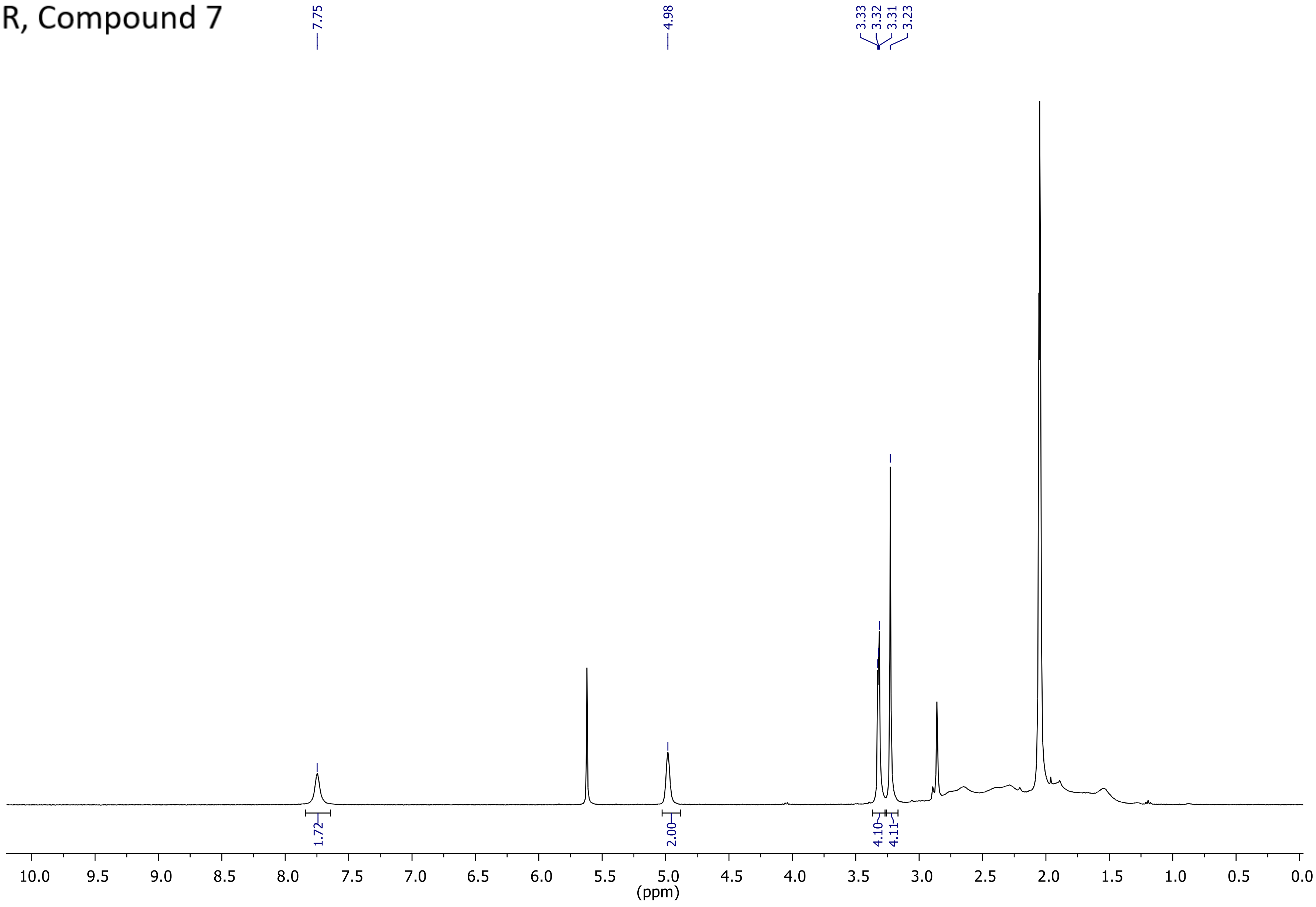

$^{11}B\{^1H\}$ NMR, Compound 7

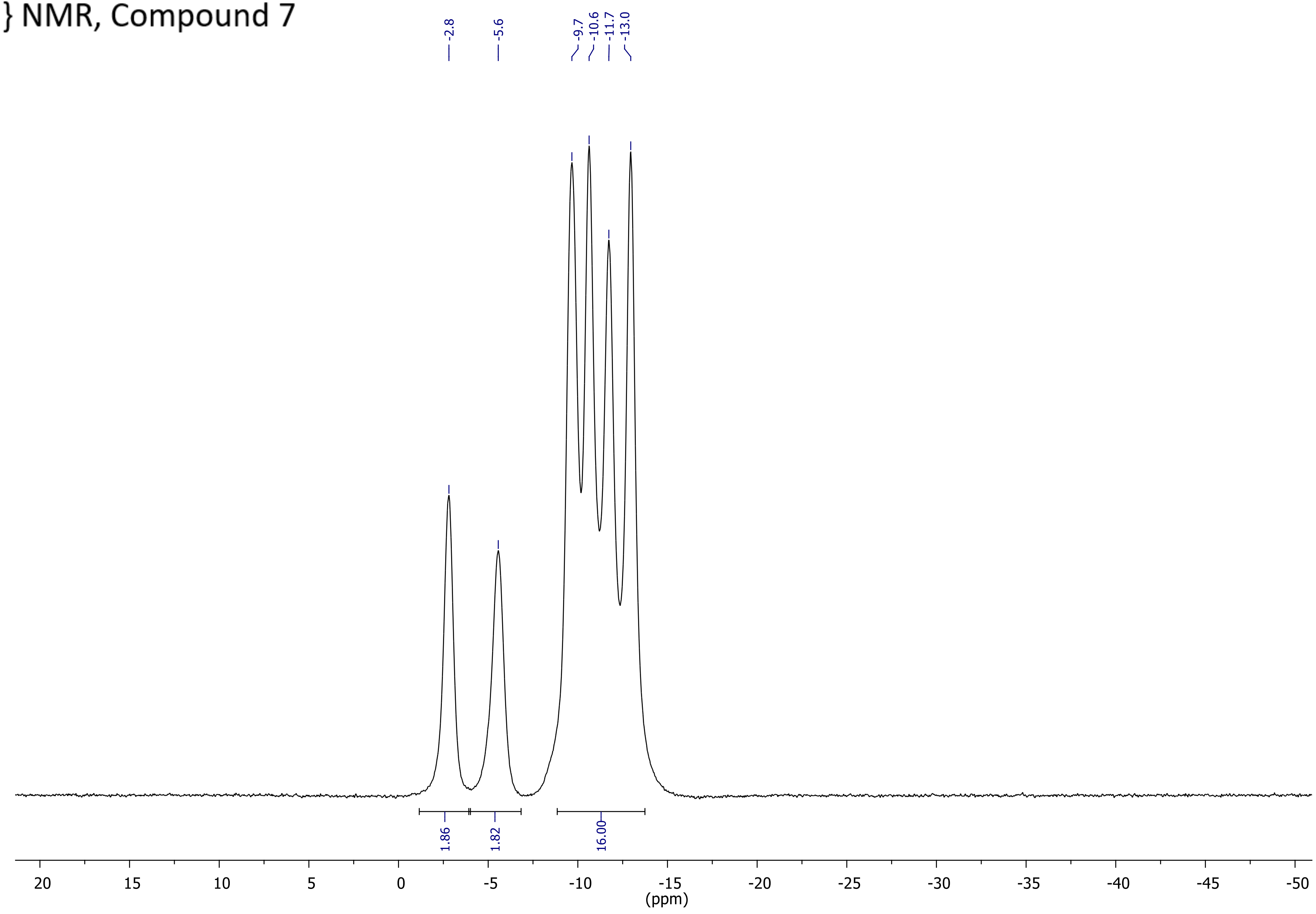

^{11}B NMR, Compound 7

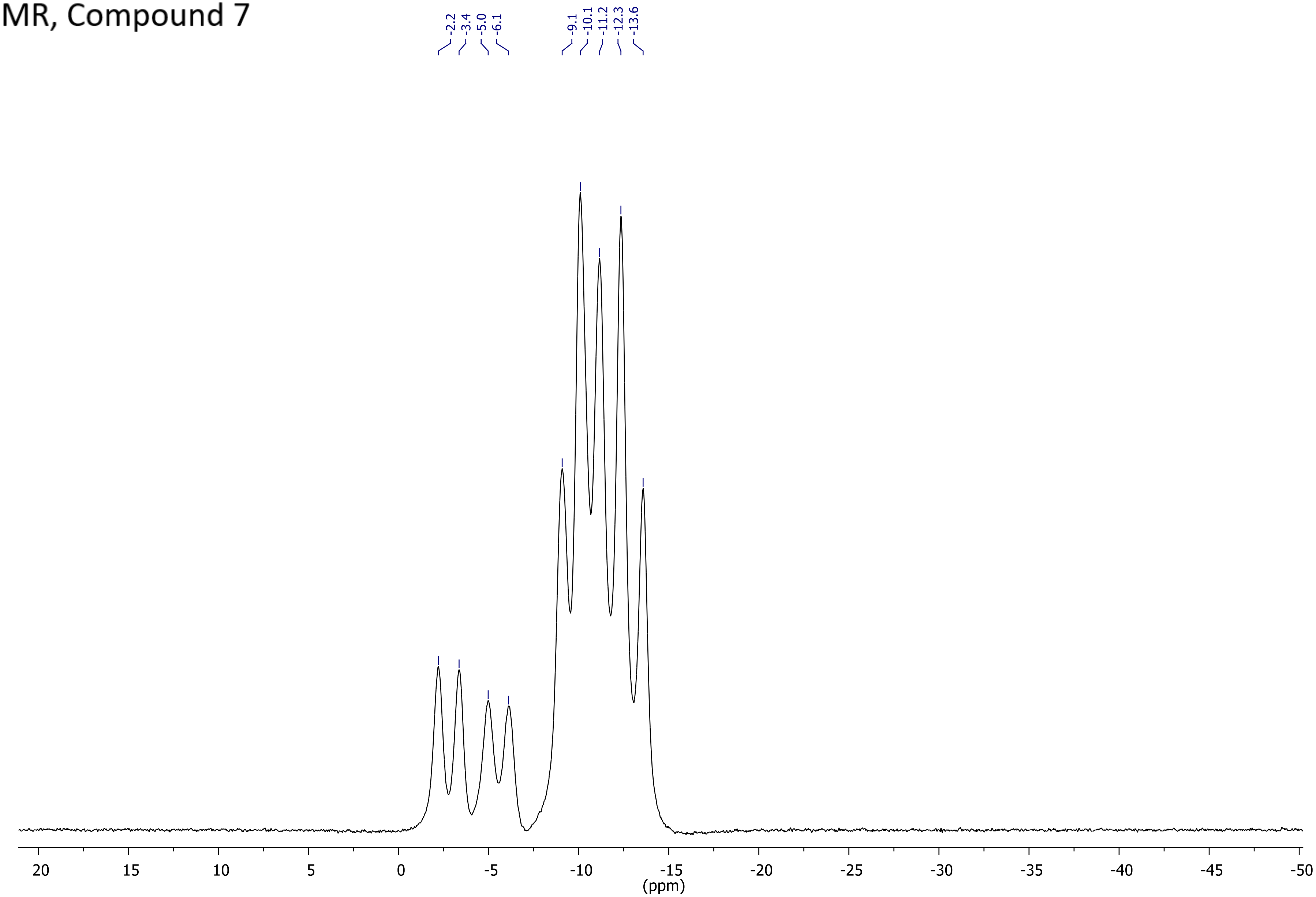

$^{13}C\{^1H\}$ NMR, Compound 7

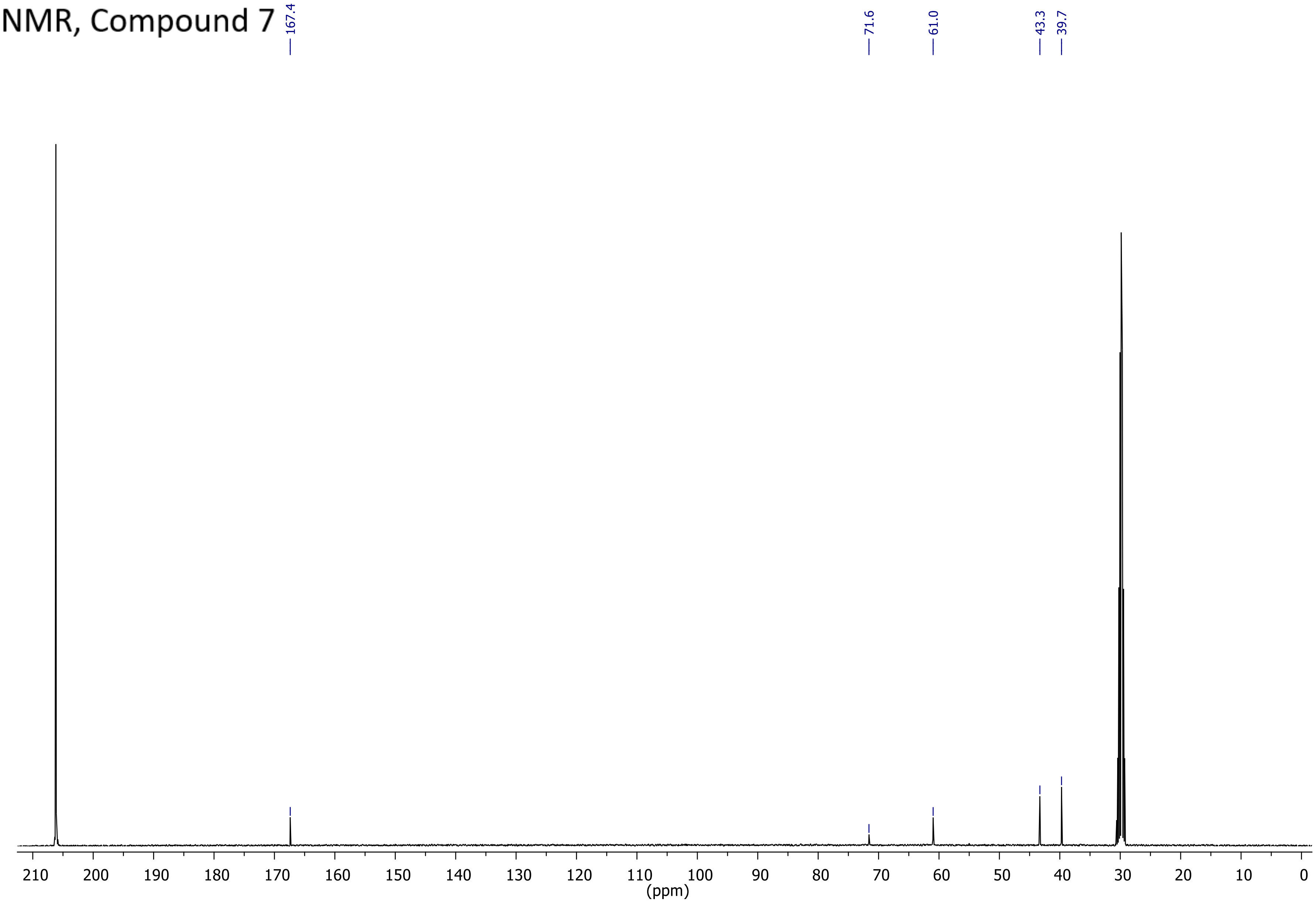

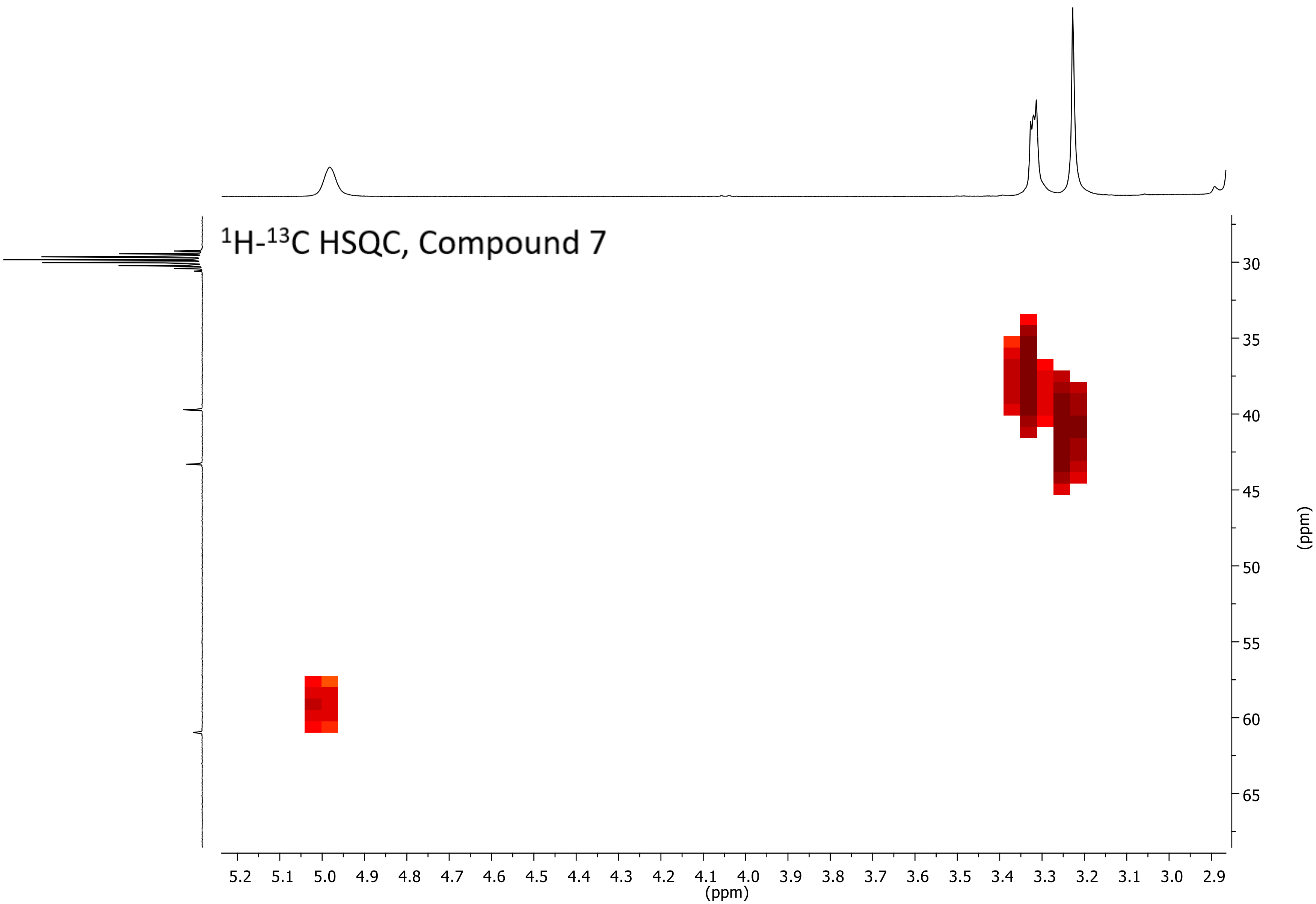

^1H-^{13}C HSQC, Compound 7
5.2
5.1
5.0
4.9
4.8
4.7
4.6
4.5
4.4
4.3
4.2
4.1
4.0
3.9
3.8
3.7
3.6
3.5
3.4
3.3
3.2
3.1
3.0
2.9
(ppm)
30
35
40
45
50
55
60
65
(ppm)

^{1}H NMR, $(Me_3NH)_2[8]$

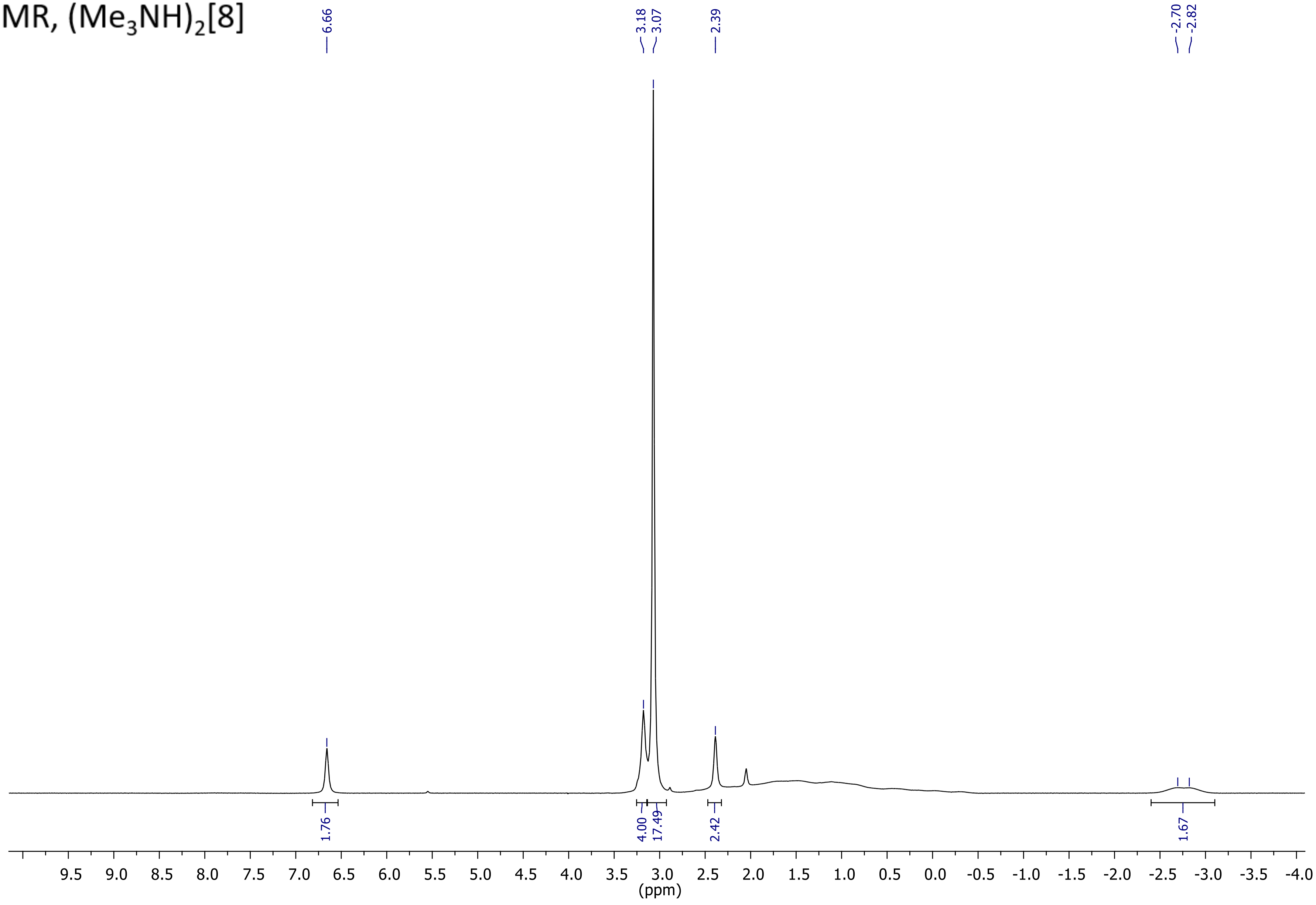

$^{11}B\{^{1}H\}$ NMR, $(Me_3NH)_2[8]$

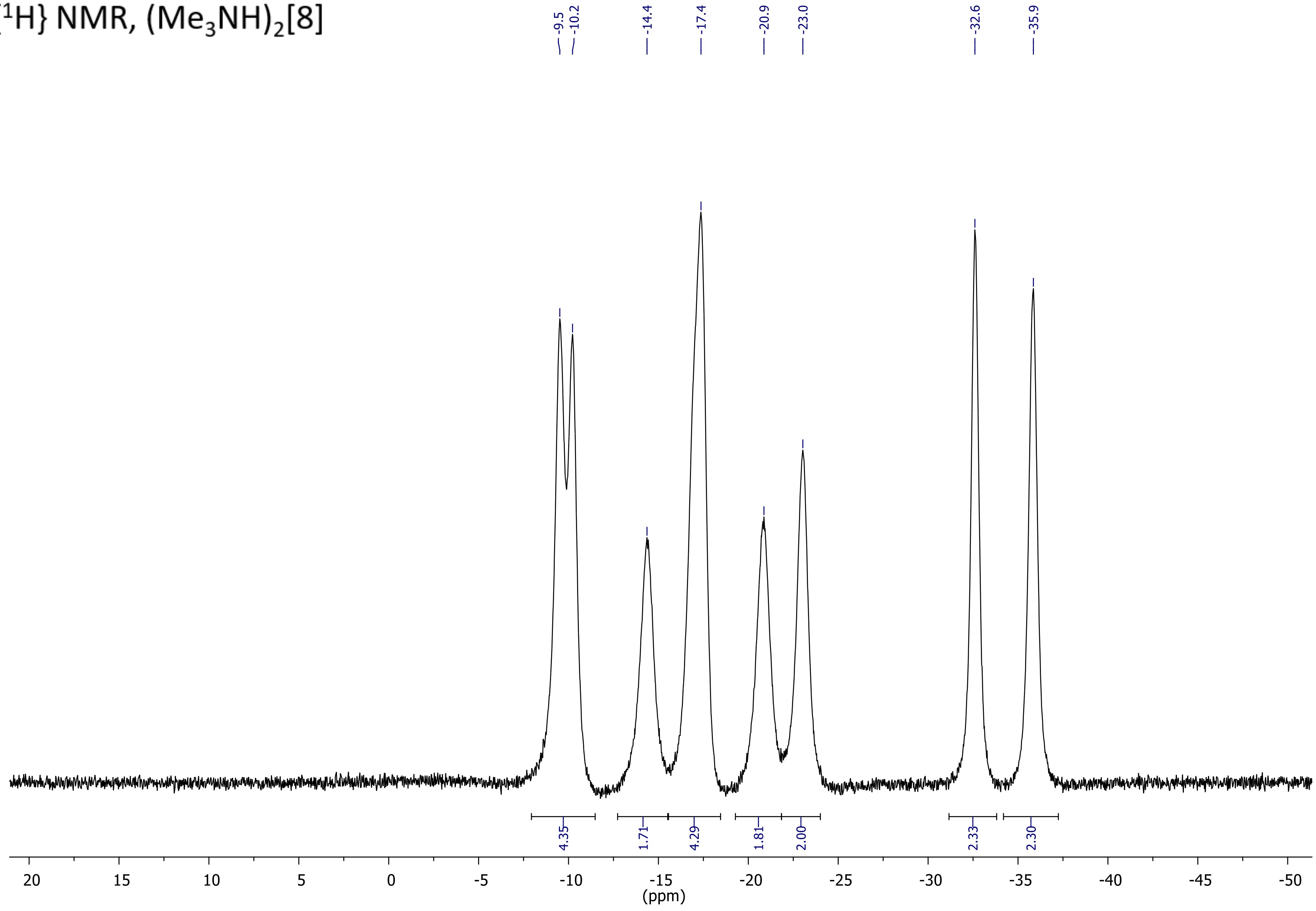

^{11}B NMR, $(Me_3NH)_2[8]$

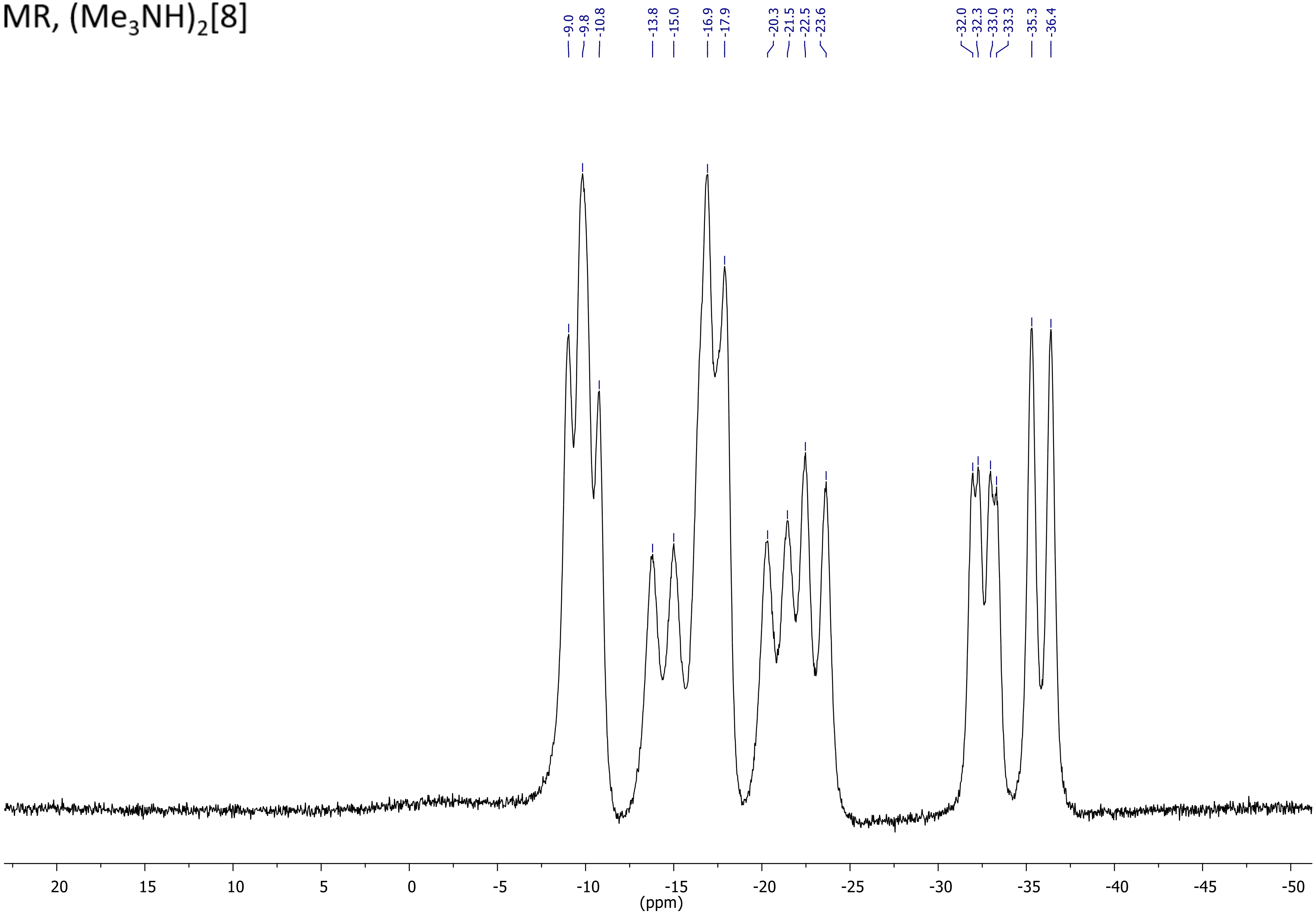

$^{13}C\{^{1}H\}$ NMR, $(Me_3NH)_2$[8]

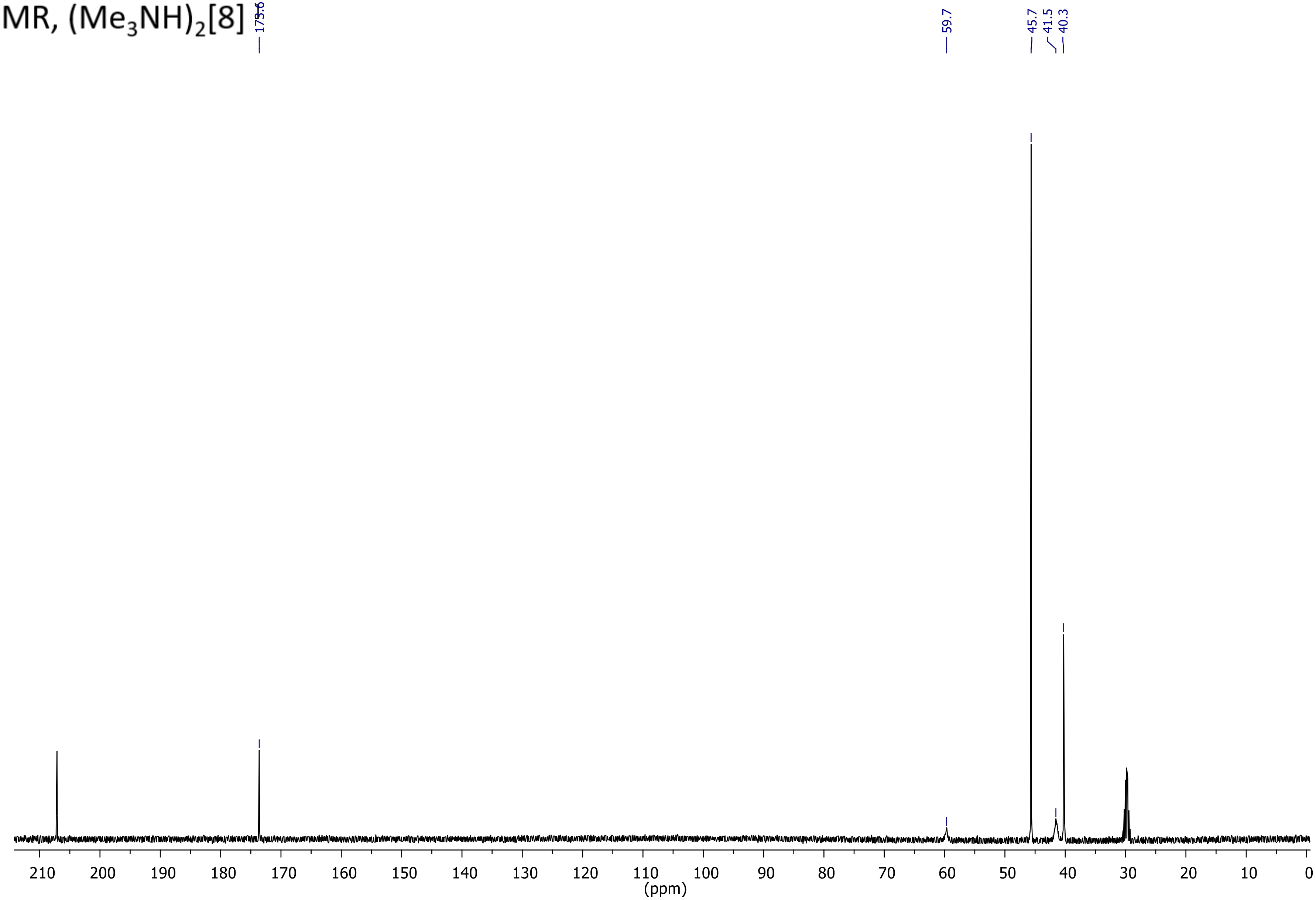

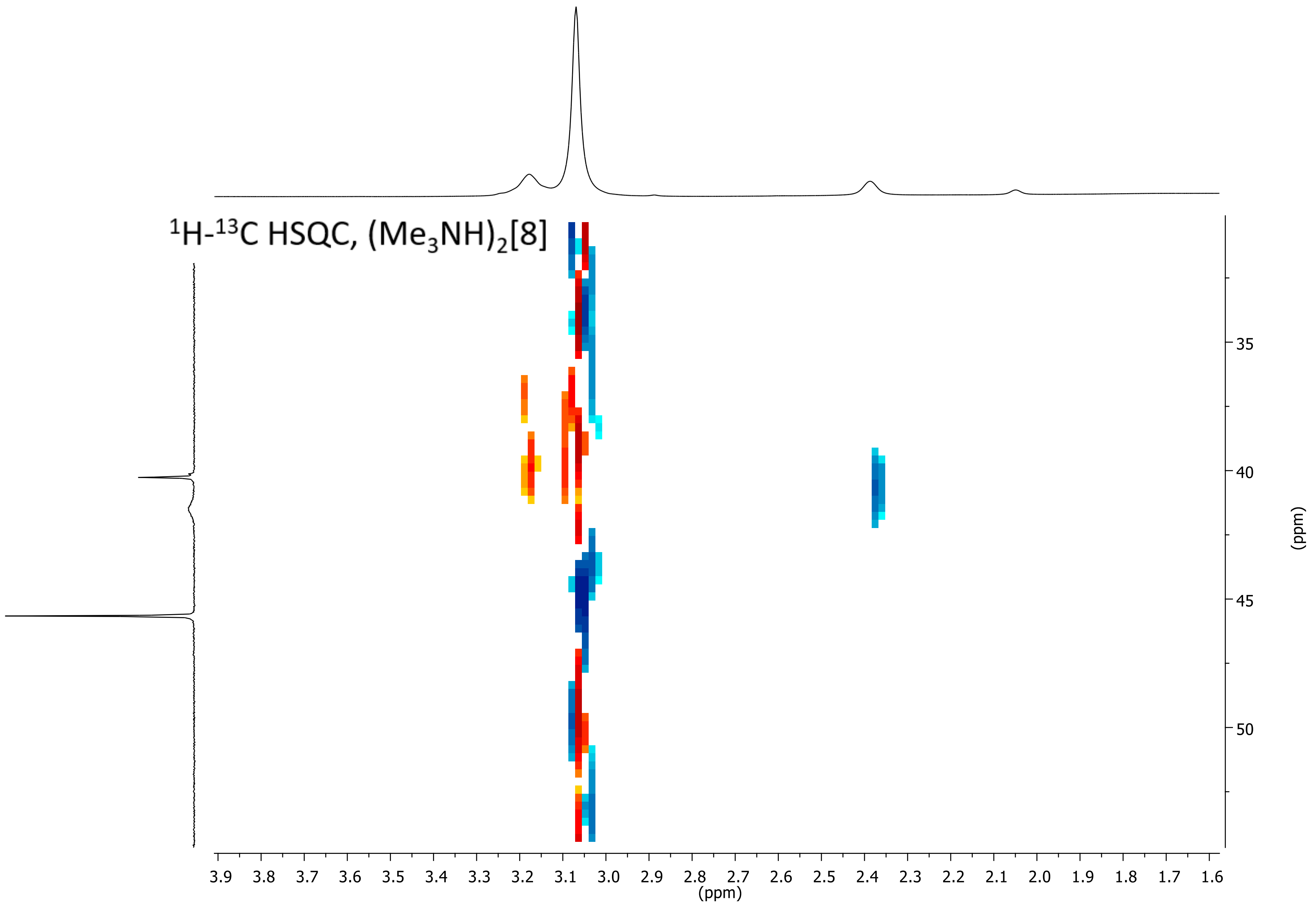

^1H-^{13}C HSQC, $(Me_3NH)_2[8]$
35
40
45
50
(ppm)
3.9 3.8 3.7 3.6 3.5 3.4 3.3 3.2 3.1 3.0 2.9 2.8 2.7 2.6 2.5 2.4 2.3 2.2 2.1 2.0 1.9 1.8 1.7 1.6
(ppm)

^1H NMR, $(Me_3NH)_2[9]$

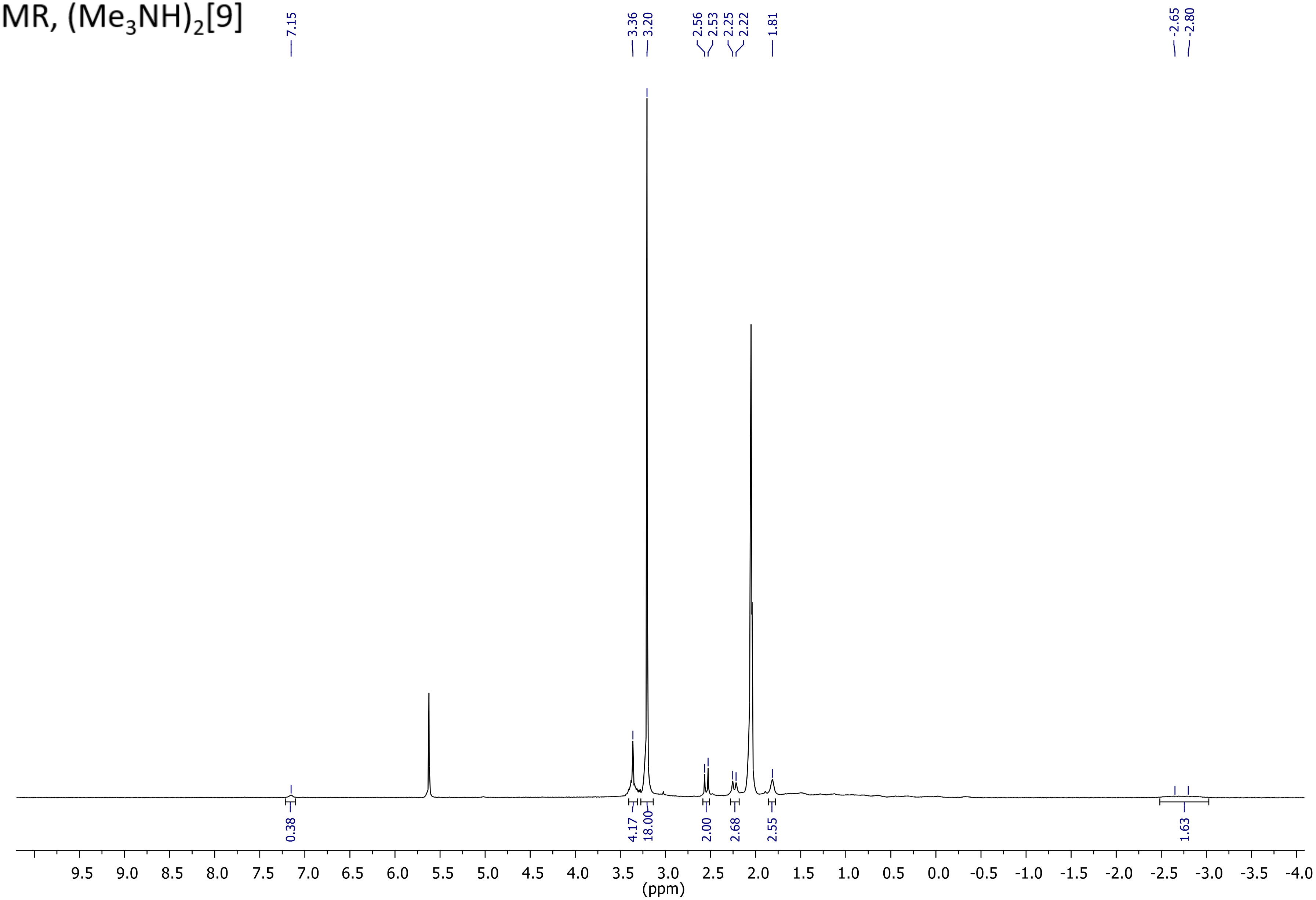

$^{11}B\{^{1}H\}$ NMR, $(Me_3NH)_2[9]$

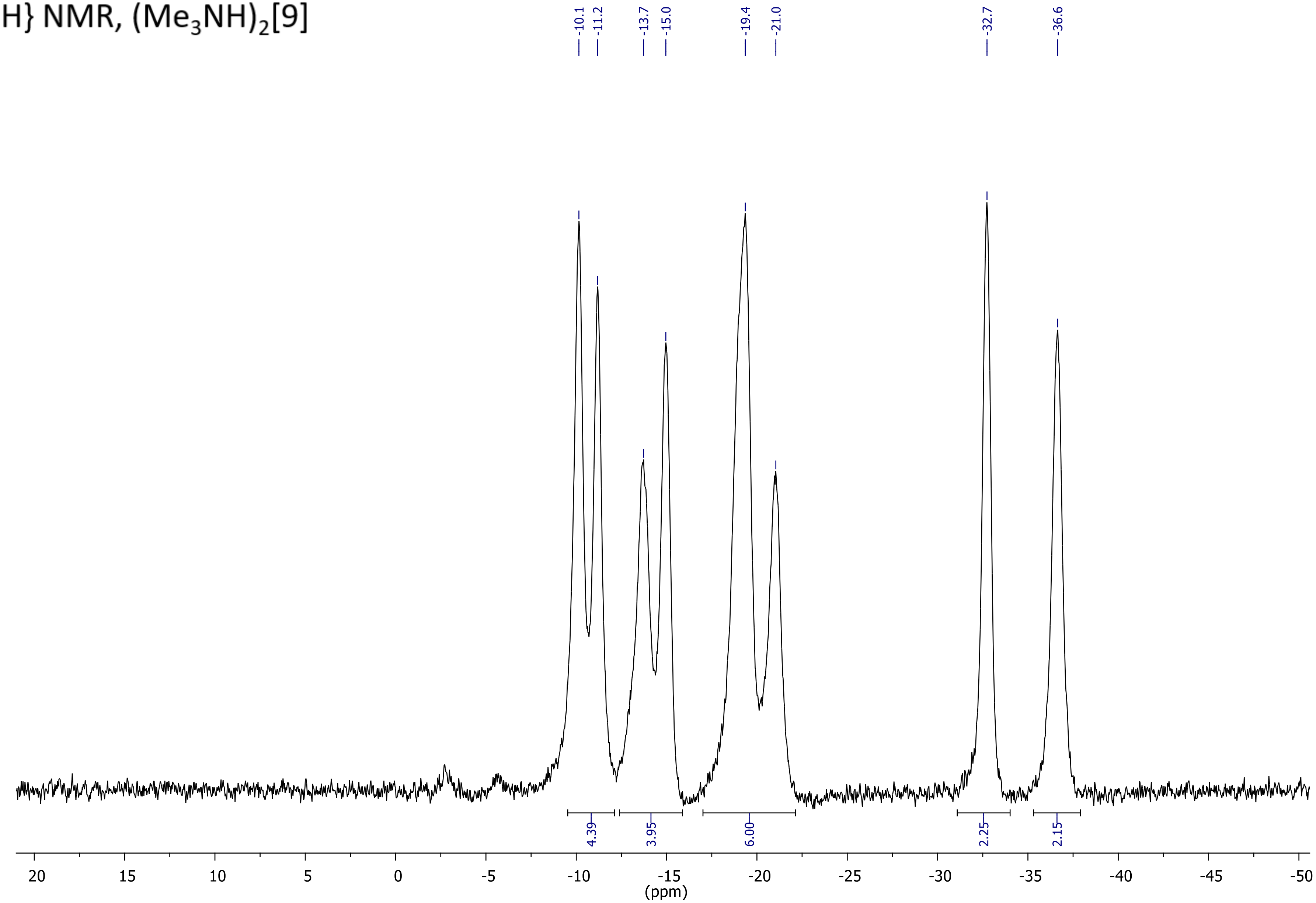

^{11}B NMR, $(Me_3NH)_2[9]$

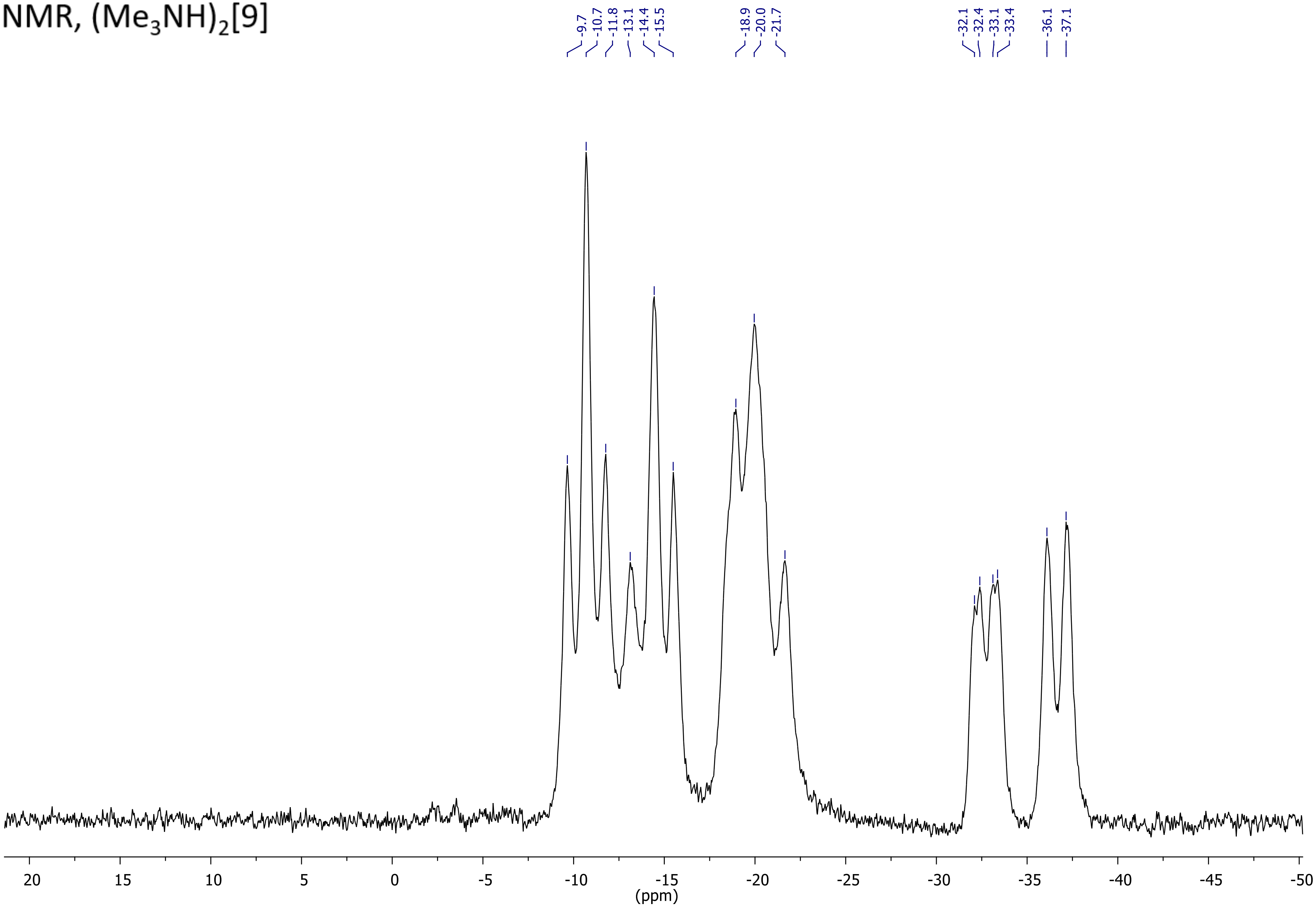

^1H NMR, $Cs_2[9]$

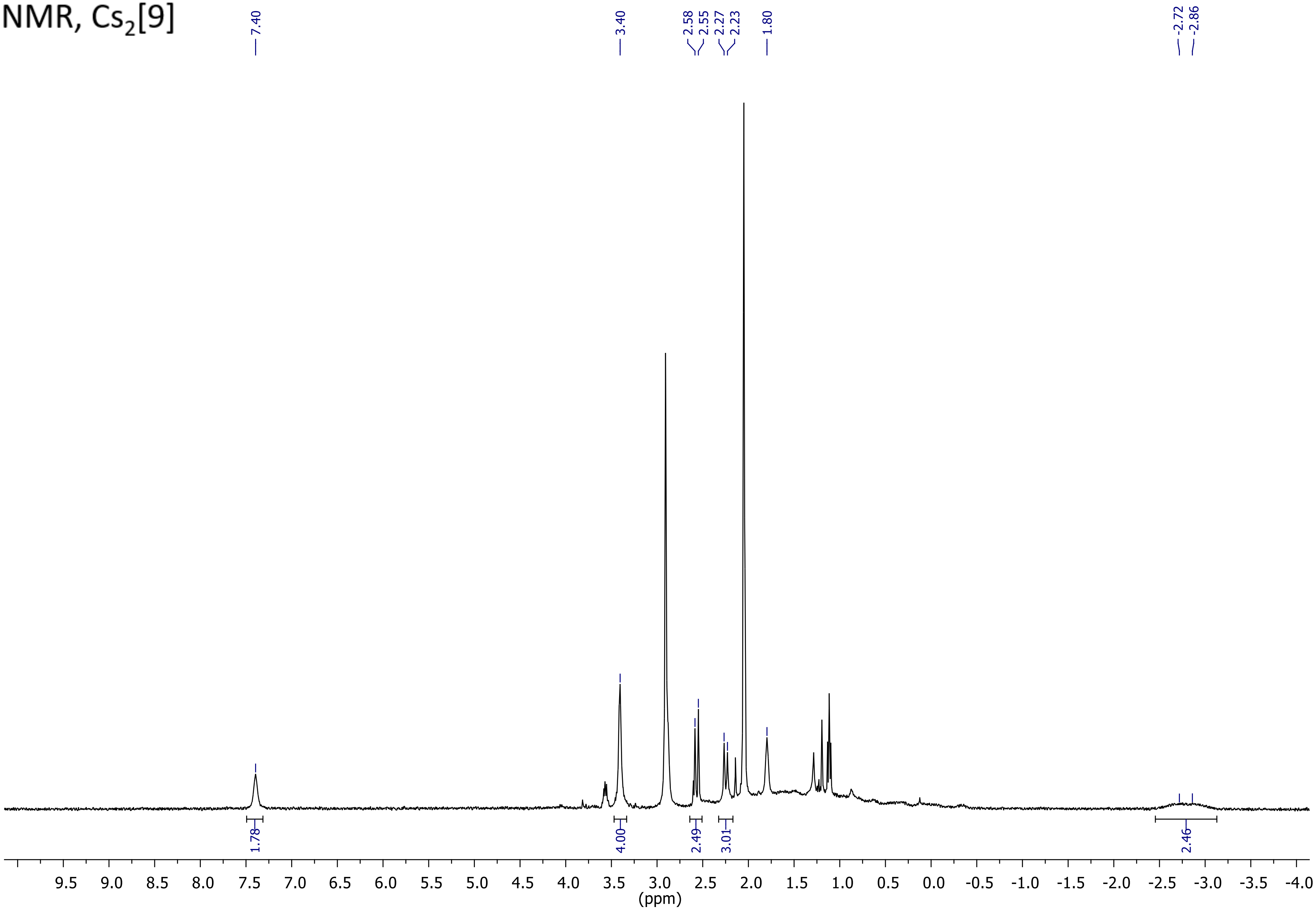

7.40
3.40
2.58
2.55
2.27
2.23
1.80
-2.72
-2.86
1.78
4.00
2.49
3.01
2.46
9.5 9.0 8.5 8.0 7.5 7.0 6.5 6.0 5.5 5.0 4.5 4.0 3.5 3.0 2.5 2.0 1.5 1.0 0.5 0.0 -0.5 -1.0 -1.5 -2.0 -2.5 -3.0 -3.5 -4.0
(ppm)

$^{11}B\{^{1}H\}$ NMR, $Cs_2[9]$

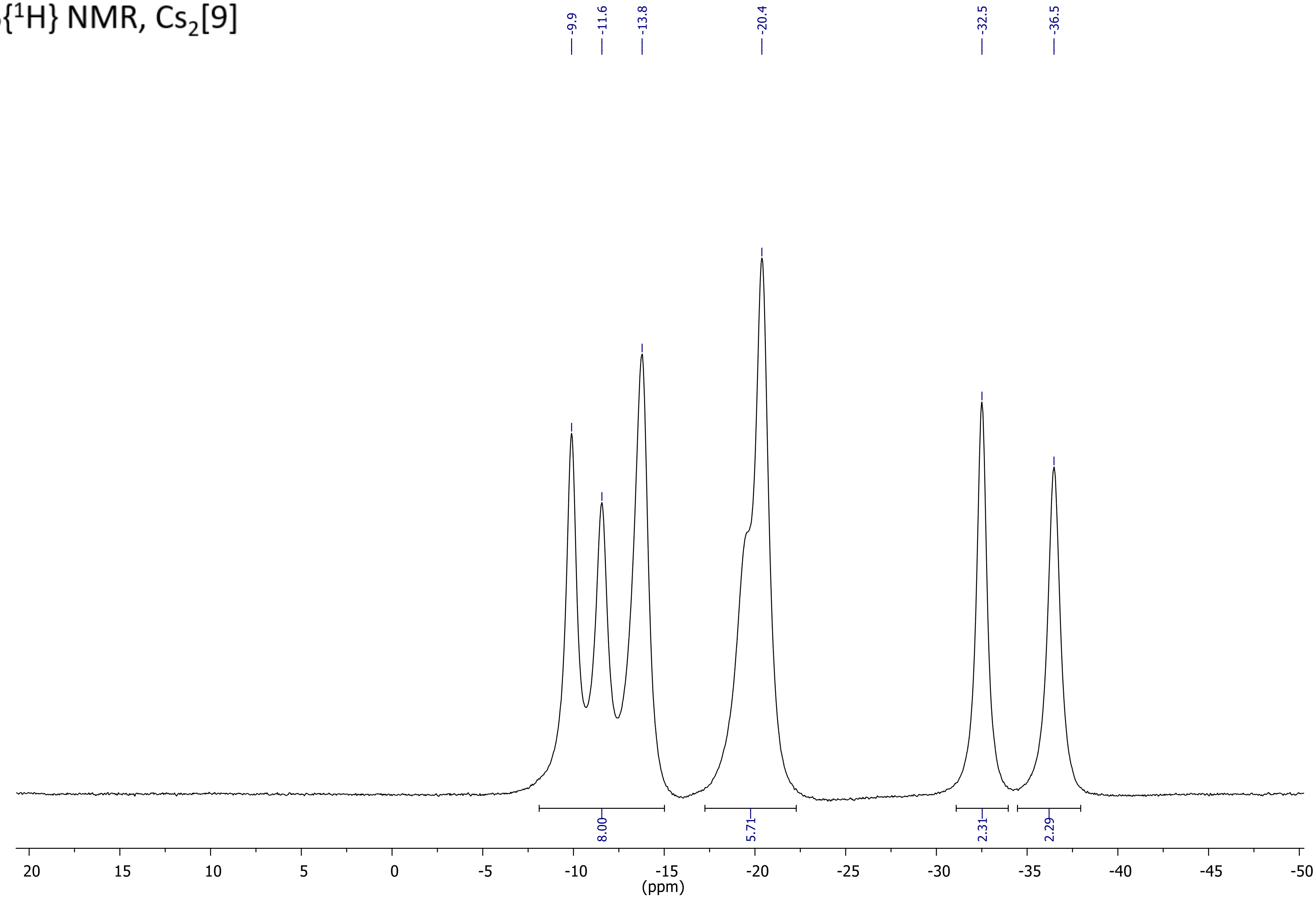

^{11}B NMR, $Cs_2[9]$

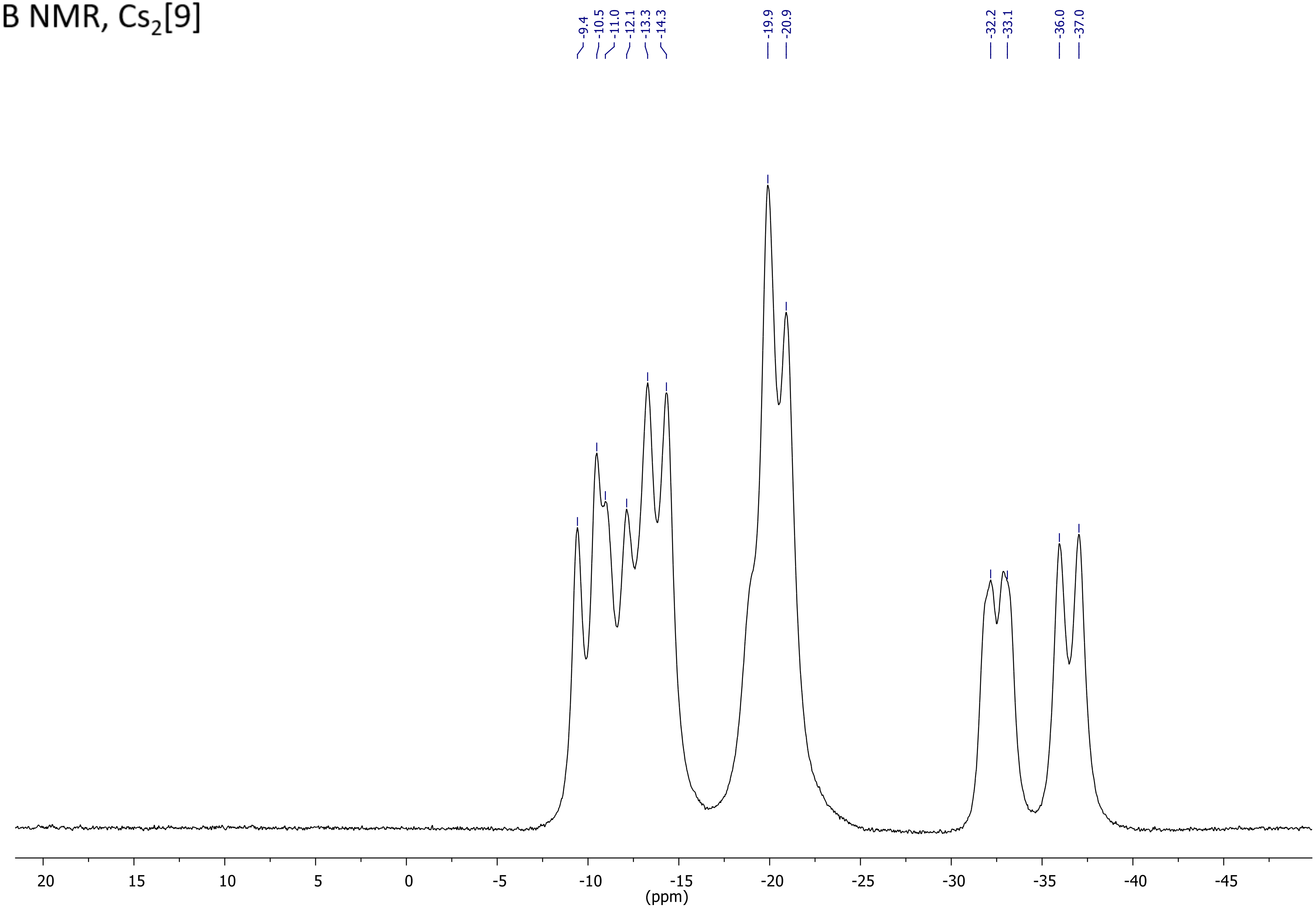
-9.4
-10.5
-11.0
-12.1
-13.3
-14.3
-19.9
-20.9
-32.2
-33.1
-36.0
-37.0
20
15
10
5
0
-5
-10
-15
-20
-25
-30
-35
-40
-45
(ppm)

Table S1. Selected geometry characteristics (distance in Å, angles in deg.) of compound **5** as obtained from X-ray study and quantum chemical calculation.

Angle or contact	X-ray	Calculation
C2-C1-C3-N1	166.3(2)	-177.5
C1-C3-N1-C4	179.2(2)	-174.6
C3-N1-C4-C4’	-117.7(2)	-86.3
N1-C4-C4’-N1’	63.6(2)	68.6
C3’-N1’C4’-C4	-119.6(2)	176.3
C1’-C3’-N1’-C4’	176.4(2)	-179.9
C2’-C1’-C3’-N1’	170.4(2)	178.3
B3-H3…H4’-B4’	2.60	2.86
B4-H4…H3’-B3’	2.51	–
N1-H1…O1’	intermol.	–
H…O	2.09	–
N…O	2.860(2)	–
<NHO	146	–
N1’-H1’…O1	intermol.	intramol.
H…O	2.12	2.06
N…O	2.899(2)	2.937
<NHO	147	144

Table S2. Energies (respective to the most favored isomer in kcal/mol) and principal geometry of $[Gd(C_2B_9H_{11})_2(DME)]^-$ rotamers.

	1	2	3	4	5	6	7	8	9
ΔE	13.4	5.8	3.0	6.4	5.3	0.0	3.1	7.4	3.1
D1	27.24	-14.25	-15.39	13.21	30.30	165.22	179.28	87.22	165.93
D2	8.69	125.28	-164.85	-127.32	-152.45	163.30	-145.98	125.03	-125.14
D3	35.93	111.03	-180.24	-114.11	-122.15	-31.48	33.30	-147.75	40.79

D1 = centroid O…O and centroid C-C of first cage: $cent_{OO}$-Gd-B_{ax}-$cent_{CC}$ dihedral.
D2 = centroid O…O and centroid C'-C' of second cage: $cent_{OO}$-Gd-B'$_{ax}$-$cent_{C'C'}$ dihedral.
D3 = sum of D1 and D2.

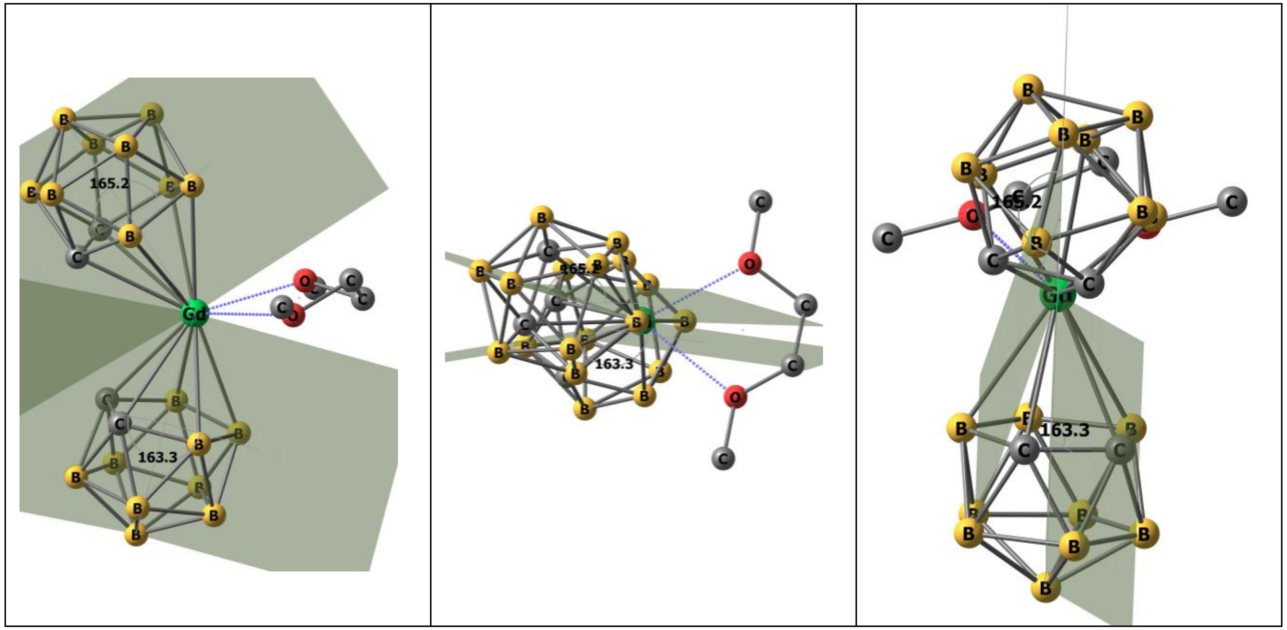

Figure S1. Optimized geometry of the most favored isomer of $[Gd(C_2B_9H_{11})_2(DME)]^-$ (three projections). Dihedrals D1 and D2 projected as olive planes, hydrogen atoms omitted for clarity.
